# Integrated biophysical assessment and erosion hotspot mapping for land degradation prioritization in the Megele washa watershed, Ethiopia

**DOI:** 10.1038/s41598-026-49658-x

**Published:** 2026-04-21

**Authors:** Kebede Bekele Atlaw, Ayele Desalegn Woldemariam, Tilahun Getachew Abebe, Getacher Kassa Mitiku, Belihu Nigatu Gorfie, Lisanu Getaneh

**Affiliations:** Amhara Agricultural Research Institute, Debre Berhan Agricultural Research Center, P.O. Box 112, Debre Berhan, Ethiopia

**Keywords:** Land use, Slope classification, Soil fertility, Soil erosion, Google Earth Engine, Geostatistical analysis, Ecology, Ecology, Environmental sciences

## Abstract

Land degradation remains a critical environmental and agricultural challenge in the Ethiopian highlands due to steep terrain and intensive cultivation. This study conducted an integrated assessment of the Megele Washa experimental Watershed using advanced geospatial techniques and R software. To achieve this, the research combined biophysical characterization (agro ecology, slope, land use/land cover and field-based soil quality evaluation) and soil erosion estimation through the Revised Universal Soil Loss Equation (RUSLE) with hotspot identification of the greatest risk. Analysis confirmed that 90% of the watershed falls within the Dega agro-ecology, while nearly half (47%) of the watershed is characterized by steep slopes (> 15%). Agricultural land dominates the watershed (58.1%), with 49% of steep areas actively under cultivation and 11.5% of the watershed classified as severely degraded land. Furthermore, soil physicochemical analysis indicated clay-dominated textures and widespread fertility decline, evidenced by spatially low levels of Total Nitrogen (TN) and Organic Carbon (OC), while pH and EC were within non-constraining ranges. Crucially, spatial analysis estimated an average annual soil loss of 26.5 t ha−1yr − 1, significantly beyond the tolerable limit, with localized losses reaching a maximum of 164.5 t ha−1yr − 1. While 78% of the area faces low-to-moderate risk, a significant 22% of the watershed is highly degraded (≥ 20 t ha−1yr − 1), constituting the critical erosion hotspots. To reduce the current average soil loss below the tolerable limit, these hotspots require targeted interventions. Implementing afforestation on steep slopes, rehabilitating degraded lands, and applying Integrated Soil Fertility Management are essential steps to restore ecological stability and enhance land productivity.

## Introduction

Ethiopia is one of the Well-endowed countries in sub-Saharan Africa in terms of natural resources and valuable diversity in the production environment. Its location in the tropics, combined with impressive altitudinal variations within short ranges, allows the country to enjoy both temperate and tropical climates, and this gives a wealth of biophysical resources, including rich biodiversity, relatively fertile soils, and huge fresh water resources. For millennia, this rich natural resource base has been serving as the foundation for agricultural development and for meeting the basic needs of millions of rural people in the country (Zeleke et al., 2006;^75^.

Despite this endowment, Ethiopia faces interlinked and reinforcing problems of land degradation and extreme poverty. This is further aggravated by high population pressure, climatic variability, top-down planning systems, lack of appropriate and/or poor implementation of polices and strategies, limited use of sustainable land management practices, limited capacity of planners, researchers, and land users, as well as frequent institutional restructuring^46^. Earlier studies estimated a topsoil loss of 1.9 billion tons annually (FAO,^23^, while more recent estimates indicate that the country loses approximately 1 to over 1.5 billion tonnes of topsoil each year, leading to agricultural productivity declines of up to 10% and national economic losses exceeding $1 billion annually^69,75^. The economic consequences of land degradation in Ethiopia range from 2% to 6.75% of the agricultural GDP (AGDP), reflecting reduced productivity, loss of ecosystem services, and increased costs for land rehabilitation^87^.

These degradation processes severely reduce agricultural productivity and heighten the vulnerability of rural communities to food insecurity. Sustainable Land Management (SLM) provides the essential framework for mitigation, yet its effectiveness depends on comprehensive baseline biophysical characterization that captures the spatial variability and ecological limitations of the landscape^1,44^. Without such a foundation, interventions are often generalized, poorly targeted, and ultimately unsustainable. For instance, recent evidence from the Ethiopian highlands shows that isolated structural measures, such as terracing, are insufficient to halt nutrient depletion; effective SLM instead requires integrating structural, vegetative, and agronomic practices alongside local knowledge and controlled grazing^17^.

Globally, integrated, soil-centered assessments are recognized as critical for achieving Land Degradation Neutrality (LDN) and promoting climate-resilient landscape restoration (SDG 15.3). Spatial modeling is increasingly vital in this regard, allowing decision-makers to pinpoint highly vulnerable hotspots often cultivated and bare lands and rank sub-watersheds to prioritize urgent SLM interventions under limited resources^37^. However, research in Ethiopia has historically focused on isolated aspects of degradation^66^. Recognizing that soil lies at the center of the land–water–vegetation continuum, integrating the effects of land use, slope, and altitude, it provides a unifying lens through which degradation drivers can be quantified and managed, and serves as a key indicator of ecosystem function, productivity, and erosion risk^65,68^.

Spatially explicit assessments of soil and landscape conditions are therefore indispensable for identifying degraded zones, mapping fertility gradients, and prioritizing interventions^65^;^68^. Recent studies in the Ethiopian highlands have emphasized the critical roles of terrain slope, land use/land cover dynamics, and soil physicochemical attributes in influencing soil erosion, fertility variation, and land degradation patterns^25,30^. Expansion of croplands into steep slopes that reflects land use change has been identified as a major driver of erosion and soil nutrient depletion in fragile ecosystems^86^. Targeting these specific drivers with integrated scalable solutions, such as pairing physical bunds with agroforestry, has proven capable of reducing soil loss by up to 60%^69^. Similarly, elevation-based agro-ecological gradients strongly influence soil formation processes, organic matter accumulation, and moisture regimes, highlighting the importance of altitude-based agro-ecological mapping for soil-based biophysical characterization^1^.

In the Megele Washa Watershed, which serves as an experimental site, this study is designed as a baseline biophysical assessment to inform subsequent SLM interventions. While broader biophysical parameters such as biodiversity and surface hydrology are important, they were excluded from this specific assessment. Instead, terrain, land cover, and soil properties were prioritized because they act as the primary physical drivers of topsoil loss and provide the explicit spatial, agronomic, and engineering data strictly required to design SLM interventions (Morgan, 2005;^30^. Consequently, these selected variables yield the most actionable baseline for prioritizing hotspot areas, optimizing resource allocation, and monitoring restoration success over time. As a complement to the biophysical assessment, socioeconomic studies were conducted previously in the watershed, used to contextualize land use practices, resource dependence, and community vulnerability.

The study provides a replicable framework for watershed-scale biophysical assessment rather than its local application. Unlike previous studies that focus primarily on soil loss estimation, this research integrates soil quality indicators, altitude-based agro-ecological zonation, and land degradation drivers with erosion hotspot mapping to support comprehensive decision-making.

This study provides a replicable and methodologically enhanced framework for watershed-scale biophysical assessment. It advances beyond conventional GIS-based RUSLE applications by implementing the model within Google Earth Engine (GEE), a computationally efficient approach that remains highly underutilized in the Ethiopian highlands. Furthermore, unlike previous studies that focus primarily on quantifying soil loss, this research provides a holistic, decision-oriented analysis. It integrates erosion hotspot mapping with slope characterization, soil quality indicators, altitude-based agro-ecological zonation, and land use/land cover dynamics. Most notably, this study introduces a spatial prioritization approach that explicitly links erosion severity with soil conditions and landscape characteristics. This allows for the targeted, evidence-based selection of Sustainable Land Management (SLM) interventions^81^. Ultimately, this integrated framework improves reproducibility and practical applicability, bridging the gap between erosion modeling and climate-resilient conservation planning.

## Materials and methods

### Description of Study Area

**Fig. 1:**
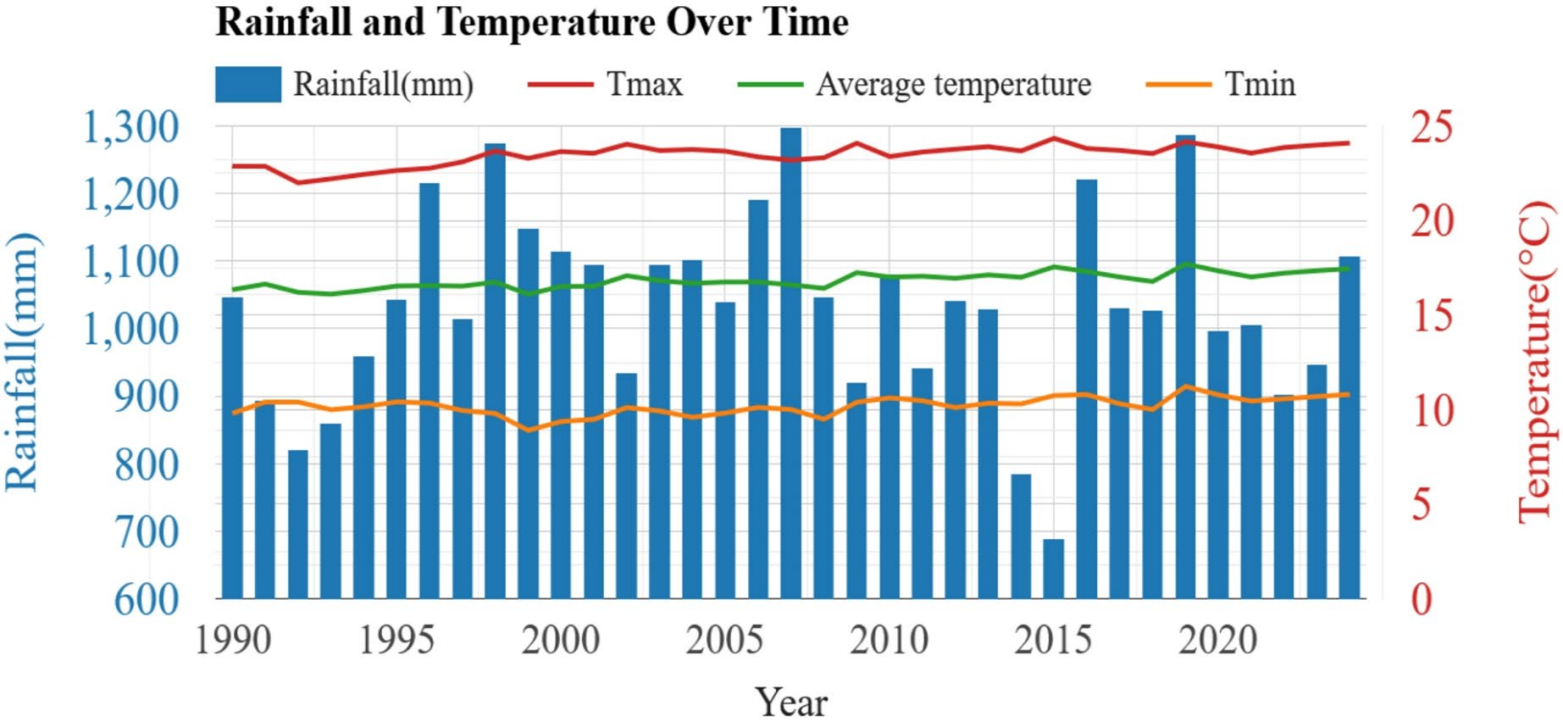
Rainfall and Temperature Patterns of the Study Area.

**Fig. 2:**
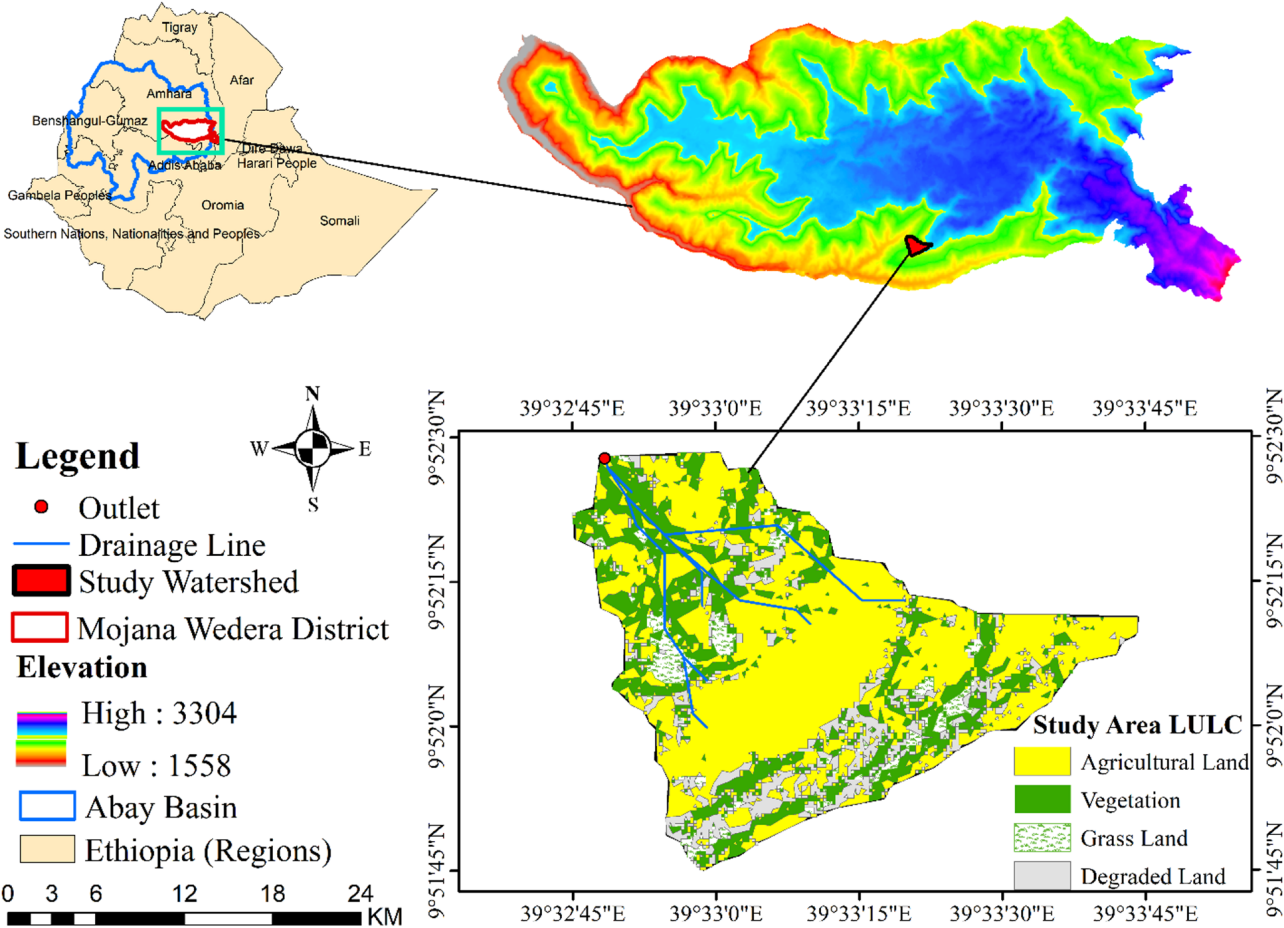
Map of the study watershed.

The study was conducted at the Megele Washa watershed (Fig. 2), found in Mojana Wodera District, Amhara Regional State, situated 225 km northwest of Addis Ababa. The watershed area covers 116 ha, which is located at 39°32’ 07” to 39°34’11” E longitude and 09°51’41” to 09°53’35” N latitude. The elevation of the watershed ranges from 2210 to 2682 m above sea level. Based on climatic records, the watershed receives a mean annual rainfall of 1,035 mm, with historical annual fluctuations ranging from 689 mm to 1,297 mm. The area experiences temperatures ranging from an absolute minimum of 8.9 °C to a maximum of 24.35 °C, with a mean annual temperature of 16.8 °C (Fig. 1). Agriculture is the major land use and land cover (LULC) type in the area. Major crop types growing are cereals (wheat, teff, chickpea, and grass pea), vegetables, and root crops (onion, garlic, cabbage), and clay texture-dominated soil is the major soil type. Small-scale agriculture and animal husbandry were the common farming systems in the watershed.

### Data to be collected and analysis methods

This study employed a multistage methodology that integrated field observation, laboratory analysis, and advanced geospatial techniques to establish a baseline for the Megele washa experimental watershed interventions. The study focused on key parameters, including Watershed delineation, agro ecology and slope classification, current land use and land cover mapping, soil physicochemical properties analysis, and soil loss estimation with hotspot identification.

### Watershed delineation, agro-ecology, and slope classification

The initial geographical definition involved watershed delineation and the extraction of crucial topographic factors. BY using the Hydrology tools in ArcGIS 10.8, the watershed boundary and stream network were accurately delineated based on the digital elevation model (DEM) (SRTM (DEM) 30 m Resolution). From the DEM, agro-ecology was classified into altitudinal zones based on elevation to reflect ecological variations influencing land use and crop suitability^35^. Slope classification was also conducted from the DEM in the ArcGIS environment to categorize terrain steepness, which is critical for assessing erosion risk and appropriate conservation practices^41^.

### Land use/land cover (LULC) classification

Land use land cover (LULC) classified using Google Earth Engine (GEE) cloud computing platform using Sentinel-2 Multispectral Instrument (MSI) imagery 2024 at 10 m spatial resolution via its JavaScript Application Programming Interface (API)^27^. GEE has recently become a popular cloud-computing platform for retrieving and analyzing geospatial data^40^. After preprocessing steps, including cloud masking and temporal compositing, spectral indices like the Normalized Difference Vegetation Index (NDVI) were derived to enhance class separability^63^. A supervised classification approach was adopted using the Random Forest (RF) algorithm^8^. Field-collected coordinate points obtained during ground surveys were utilized as training and validation samples to improve the accuracy and representativeness of the classification. The accuracy was evaluated using an error/confusion matrix with Overall Accuracy (OA), Producer’s Accuracy (PA), User’s Accuracy (UA), and the Kappa coefficient^16^, providing a reliable LULC map.

### Soil data collection and laboratory analysis

#### Sampling and field collection

**Fig. 3:**
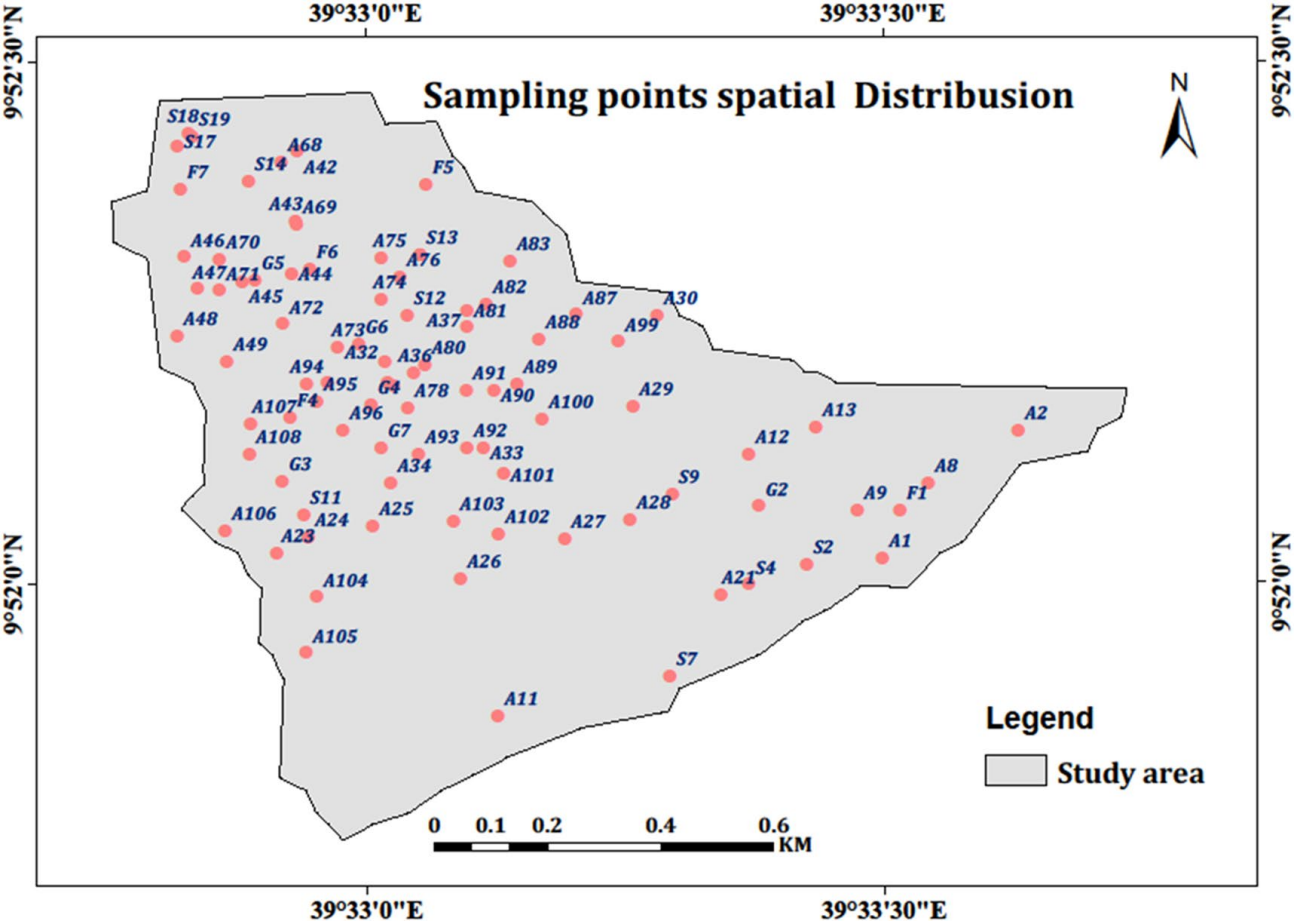
Spatial distributions of soil sampling points of the study area. *NB. The labels on the map represent major land uses: A-agricultural land*,* F-forestland*,* S-shrub*,* and G–grazing land*.

90 soil samples were collected across the 116 ha watershed, corresponding to a sampling density of approximately one sample per 1.3 ha. This relatively high density was selected to capture the spatial variability of soil properties in a topographically complex watershed and to support reliable geostatistical analysis^77^. Intentionally stratified sampling based on land use and slope variability was taken as the sampling design to capture the full range of degradation status. Sampling points were proportionally allocated across strata, and their spatial distribution is presented in Fig. 3.

Samples were collected at depths of 0–20 cm for cultivated land, and 0–30 cm for remaining land uses, using a soil auger, while undisturbed core samples were collected with a core sampler for bulk density determination. For cultivated land, the 0–20 cm depth corresponds to the standard active plow layer, which captures the zone of frequent tillage, active soil-nutrient mixing, and the primary rooting depth of annual crops^35,78^. Conversely, for other land uses a deeper 0–30 cm profile was sampled to account for undisturbed organic matter accumulation, deeper humification processes, and the permanent rooting zones of perennial vegetation, aligning with standard international carbon and nutrient baseline protocols (IPCC, 2006). The use of different sampling depths may introduce minor inconsistencies when comparing absolute soil nutrient stocks, as deeper samples represent greater soil mass. Ideally, such comparisons require equivalent soil mass correction to ensure consistency^20^. However, in this study, emphasis is placed on relative differences within land use types rather than direct cross-land use comparison of absolute values. Composite samples were prepared for each sampling point and placed in clean plastic bags. The corresponding coordinates were recorded using a handheld GPS device to ensure spatial accuracy.

### Physicochemical parameters analyzed

The major parameters analyzed included: pH, Electrical Conductivity (EC), Bulk Density (BD), Cation Exchange Capacity (CEC), Exchangeable Cations (Na, K, Ca, Mg), Organic Carbon (OC), Organic Matter (OM), Available Phosphorus (AVP), Total Nitrogen (TN), Carbon to Nitrogen Ratio (C: N Ratio), and Soil Texture (Sand, Clay, and Silt).

### Laboratory methods

Soil samples underwent detailed physicochemical analysis in the laboratory to determine key parameters like Total nitrogen (TN)(Via kjeldhl method)^11^ , Organic Carbon (OC) using Loss on Ignition (LOI) method^36^ and soil texture using the modified hydrometer method, classified by USDA system^35^; USDA,^70^.

Bulk density is determined by dividing the dry mass by the core volume^4^. The EC and pH were measured with a conductivity meter and a pH meter in water (H2O) suspension in a 1:2.5 (soil: liquid) ratio, respectively. Exchangeable bases (Na, K, Mg, and Ca) were determined after extracting the soil samples with ammonium acetate (1 N NH4OAc) at pH 7.0^61^. Cation exchange capacity (CEC) was calculated titrimetrically by distillation (FAO,^23^.

### Statistical and geostatistical analysis

#### Descriptive and correlation analysis

Descriptive statistics (mean, SD, min, max, skewness, and kurtosis) were used to summarize the laboratory data in R software. Correlation analysis conducted measures the strength and direction of the relationship between two (or more) soil parameters. The Pearson correlation coefficient (r) was also employed to identify redundant parameters. This was a critical preprocessing step: strongly correlated variables were represented by a single parameter to establish the Minimum Data Set (MDS) of Soil Quality Indicators, ensuring the subsequent spatial analysis was focused and free of multicollinearity^32,77,78^. Furthermore, this analysis guided the selection of a Core Set of Informative Parameters for geostatistical mapping, enabling the identification of key interdependencies among soil properties.

### Geostatistical modeling

Ordinary Kriging (OK) was the chosen geostatistical method for spatially predicting the Soil Quality Indicator, providing the Best Linear Unbiased Predictions (BLUP) based on spatial autocorrelation^64^. Before OK, the dataset was visually inspected through histograms and box plots in ArcGIS and Microsoft Excel 2019. Data normality assessed using the Shapiro–Wilk test in R software and further verified through Normal Quantile–Quantile (Q–Q) plots. Outliers were flagged and redefined using the mean weighted method. Data transformation, trend analysis, and detrending are performed to ensure that the assumptions required for Ok were satisfied^50^. Specifically, global trends often caused by continuous elevation or slope gradients across the watershed were evaluated using trend analysis plots. When a systematic trend was identified, a detrending procedure was applied using either a first-order (linear) or second-order (quadratic) polynomial. The selection of the polynomial order was justified by evaluating goodness-of-fit metrics, choosing the lowest order necessary to remove the systematic variation, thereby ensuring the residuals met the strict stationarity assumption required for OK^26^.

The experimental semivariogram (γ(h) was calculated to quantify spatial dependence (autocorrelation), and theoretical models (Circular, Spherical, Exponential, Gaussian) were fitted using weighted least squares^77^. It is determined as follows (1)

1 $$\:\:\:\:\:{\upgamma\:}\left(\mathrm{h}\right)=\:\left(\frac{1}{2N}\left(\mathrm{h}\right)\right){\sum\:}_{i=1}^{N\left(h\right)}\left(Z\right(x_i)\:-\:Z(x_i+h\left)\right)^2$$

Where: N (h) = number of pairs of points separated by distance h. i- is the sampling number. Z(x_i_) and Z(x_i_+h) are measured values at locations x_i_ and x_i_+h, respectively.

The selection of the best-fitting theoretical variogram model was not arbitrary; it was systematically determined using a combination of goodness-of-fit measures and leave-one-out cross-validation. The model demonstrating the lowest Residual Sum of Squares (RSS) on the experimental variogram, coupled with the lowest Root Mean Square Error (RMSE) and a Mean Error (ME) closest to zero during cross-validation, was selected as the optimal model for interpolation^62^.

Key semivariogram parameters were derived as follows: the nugget (C₀) represents measurement error or microscale variations, the sill (C₀ + C) denotes the total spatial variance, and the range (a) defines the distance beyond which samples become spatially uncorrelated. The nugget-to-sill ratio (C₀ / (C₀ + C) was used to classify spatial dependence, with values < 25% indicating strong, 25–75% moderate, and > 75% weak spatial dependence^12,32,77^. However, while these thresholds provide a useful reference, they were interpreted cautiously as relative indicators rather than rigid rules. It is acknowledged that the nugget-to-sill ratio can be strongly influenced by sampling density and micro-topographic variability, such that a higher ratio reflects unresolved microscale variation rather than invalidating the overall spatial structure^53^.

The OK estimate at an unsampled location x_0_ is calculated as the weighted linear combination of observed values (2):

2 $${\rm Z_{OK}(x_0)} = \sum_n^{i=1} \lambda_i Z(x_i)$$

Where: *Z(xi)* is the observed value at location *xi*,* i* is the sampling number. λ_i_ = kriging weights determined under unbiasedness constraint.

Model performance and prediction reliability were evaluated using leave-one-out cross-validation. The accuracy indices included root mean square error (RMSE) and root mean square standardized error (RMSSE)^13,50^. A smaller RMSE relative to the data range indicates better prediction accuracy, with normalized RMSE (NRMSE) values below 10–20% considered acceptable^14^. RMSSE close to 1 suggests that the model’s estimated variances are unbiased. These were computed as follows (3&4):

3 $$\:\mathrm{R}\mathrm{M}\mathrm{S}\mathrm{E}=\frac{1}{\mathrm{n}}\sum\:_{\mathrm{i}=1}^{\mathrm{n}}\sqrt{{\left[\mathrm{Z}\left(\mathrm{x}_i\right)-\:\mathrm{Z}*\left(\mathrm{x}_i\right)\right]}^{2}}$$

4 $$\:\mathrm{R}\mathrm{M}\mathrm{S}\mathrm{S}\mathrm{E}\:=\:\sqrt{\frac{1}{\mathrm{n}}{\sum\:}_{\mathrm{i}=1}^{\mathrm{n}}\left[\frac{\left(\mathrm{Z}\left(\mathrm{x}_i\right)-\:\mathrm{Z}*\left(\mathrm{x}_i\right)\right)}{{\upsigma}_k \left(\mathrm{x}_i\right)}\right]}$$

Where Z*(x_i_) is the predicted value at location x_i_, σₖ (x_i_) is the kriging standard deviation at location x_i_.

### Soil Loss Estimation, Hotspot Prioritization, and Sensitivity Analysis

### Revised universal soil loss equation (RUSLE)

**Fig. 4:**
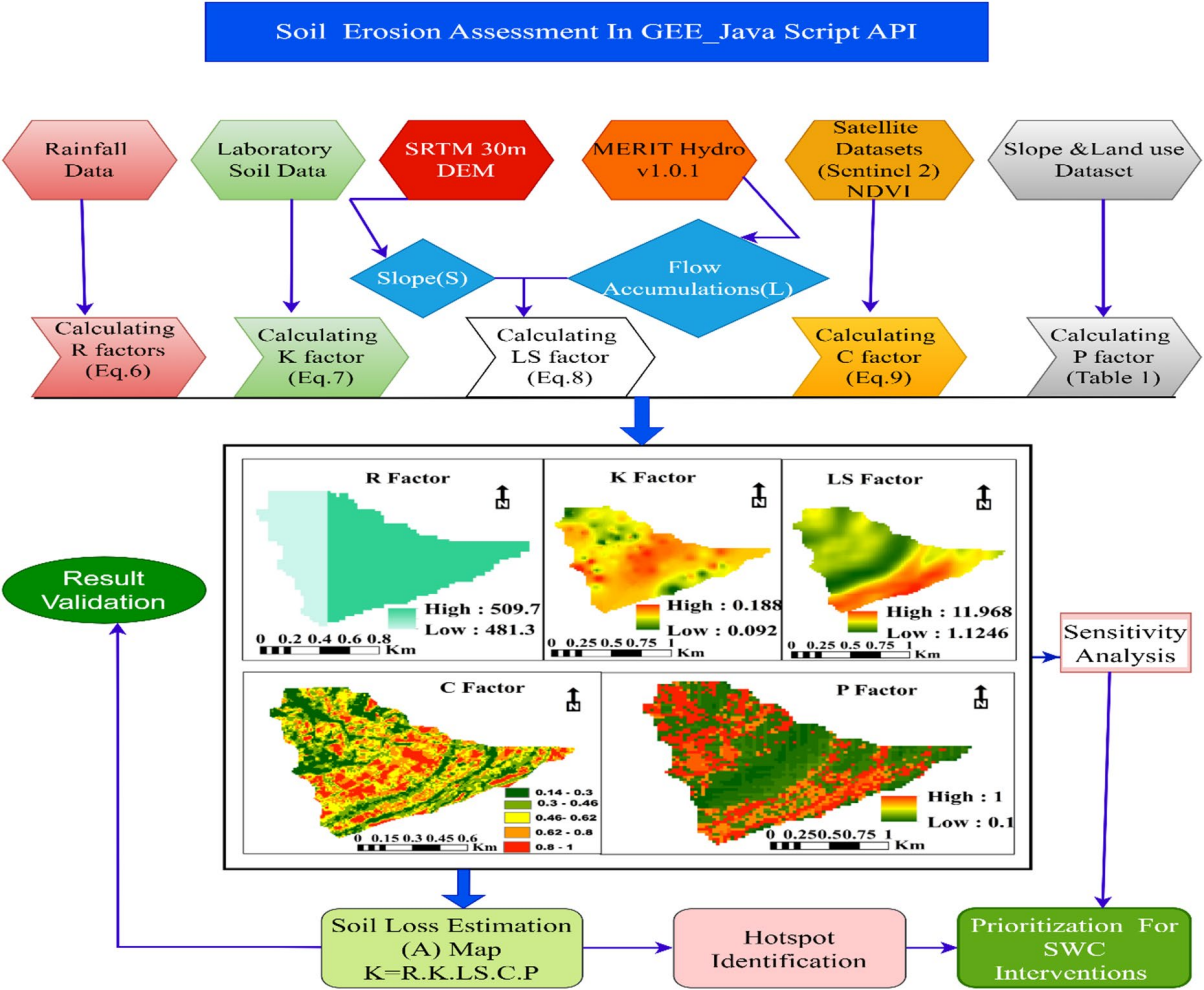
Schematic Presentation of Soil Erosion Risk Assessment Using the RUSLE Model.

Annual soil loss (A) was estimated using the Revised Universal Soil Loss Equation (RUSLE), which is an empirical model that estimates the long-term average soil loss rate by taking into account six factors that affect soil erosion. These include rainfall erosivity, soil erodibility, slope length and steepness, cover management, and conservation practices^60^. The model was implemented primarily in Google Earth Engine (GEE) with ground-based rainfall and soil data (Fig. 4). This approach is nowadays mentioned as the GEE-RUSLE framework^56^. All factor raster and the final soil loss map were exported to GIS (GeoTIFF format) for visualization and further spatial analysis. The popular RUSLE formula is presented in Eq. (5).

5 $$\:A\hspace{0.17em}=\hspace{0.17em}R\:*\:K\:*\:LS\:*\:C\:*\:P$$

Where: A is the long-term mean annual soil loss rate (t ha − 1 yr − 1), R is the rainfall erosivity factor (MJ mm ha − 1 h − 1 yr − 1), K is the soil erodibility factor (t ha h ha − 1 MJ−1 mm − 1), LS is the topographic factor (dimensionless) which combines slope length (L) and slope steepness (S), C is the cover management factor (dimensionless), and P is the support practice factor (dimensionless).

### Rainfall erosivity factors (R)

The rainfall erosivity factor (R) measures the power of rainfall to cause soil erosion. The rainfall erosivity factor (R) was calculated from long-term (1993–2024) ground-based precipitation measurements obtained from the National Meteorology Agency station (~ 19 km from the study area). Applying this point data to the entire watershed assumes a uniform rainfall distribution across the study area. However, it is acknowledged that given the watershed’s significant elevation range, relying on a station 19 km away introduces a degree of spatial uncertainty due to potential micro-orographic rainfall variations typical of the highly undulating Ethiopian highlands^31^. Such variability can influence rainfall intensity and erosivity, potentially affecting the spatial accuracy of the estimated R-factor. In the absence of a localized gauge within the watershed, this station provides the most reliable long-term temporal coverage. To minimize this uncertainty, the Ethiopian-adapted equation (Hurni et al., 2015) was utilized, as it is robustly calibrated for the regional agro-ecological conditions (Eq. 6):

6 $$\:R\hspace{0.17em}=\hspace{0.17em}0.562*P\:-8.12$$

Where P is the long-term mean annual rainfall (mm), imported into GEE as a Feature Collection, and interpolated to generate a continuous raster representing spatial rainfall erosivity across the watershed.

### Soil erodibility factor (K)

The K-factor represents the erodability of soil particles by water that is derived from laboratory-analyzed soil properties (texture and organic carbon) using the RUSLE-based formula^79^. The full mathematical expressions used to derive the K-factor and its constituent sub-factors are presented below (Eqs. 7 and 7a–7d): To ensure unit consistency with the metric rainfall erosivity factor, a standard conversion factor of 0.1317 was applied to convert the K-factor from US customary units to metric SI units (t·ha·h·ha⁻¹·MJ⁻¹·mm⁻¹)^24,59^

7 $$\:\mathrm{K}_{\mathrm{U}\mathrm{S}\mathrm{L}\mathrm{E}}\:=\:\mathrm{f}_{\mathrm{c}\mathrm{s}\mathrm{a}\mathrm{n}\mathrm{d}}\:\mathrm{*}\:\mathrm{f}_{\mathrm{c}\mathrm{i}\_\mathrm{s}\mathrm{i}}\:\mathrm{*}\:\mathrm{f}_{\mathrm{o}\mathrm{r}\mathrm{g}\mathrm{c}}\:\mathrm{*}\:\mathrm{f}_{\mathrm{h}\mathrm{i}\mathrm{s}\mathrm{a}\mathrm{n}\mathrm{d}}$$

#### Where

*f*csand is a factor that lowers the *K* indicator in soils with high coarse-sand content and increases it for soils with low sand content; *f*ci-si gives low soil erodibility factors for soils with high clay-to-silt ratios; *f*orgc reduces *K* values in soils with high organic carbon content, while *f*hisand lowers *K* values for soils with extremely high sand content.

7a $$\:\mathrm{f}\mathrm{c}\mathrm{s}\mathrm{a}\mathrm{n}\mathrm{d}\:=\left(0.2\:+\:0.3\mathrm{*}\:\mathrm{e}\mathrm{x}\mathrm{p}[-0.0256\:\mathrm{*}\:\mathrm{m}\mathrm{s}\:\mathrm{*}\:(1\:-\:\frac{{m}_{silt}}{100}\left)\right]\right)$$

7b $$\:\mathrm{f}\:\mathrm{c}\mathrm{l}\_\mathrm{s}\mathrm{i}\:=\:\left(\frac{{m}_{silt}}{{m}_{c}+{m}_{silt}}\right)^{0.3}$$

7c $$\:\mathrm{f}\mathrm{o}\mathrm{r}\mathrm{g}\mathrm{c}\:=\left(1\:-\:\frac{0.25\:\mathrm{*}\:\mathrm{o}\mathrm{r}\mathrm{g}\mathrm{C}}{(\mathrm{o}\mathrm{r}\mathrm{g}\mathrm{C}\:+\:\mathrm{e}\mathrm{x}\mathrm{p}[3.72\:- \:2.95\:\mathrm{*}\:\mathrm{o}\mathrm{r}\mathrm{g}\mathrm{C}]\:}\right)$$

7d $$\:\mathrm{f}\mathrm{h}\mathrm{i}\mathrm{s}\mathrm{a}\mathrm{n}\mathrm{d}\:=\left(1-\:\frac{0.7*(1-\frac{{m}_{s}}{100})}{1-\frac{{m}_{s}}{100}+\mathrm{e}\mathrm{x}\mathrm{p}[-5.51+22.9*(1-\frac{{m}_{s}}{100})}\right)$$

  

where, *f*ms is the sand fraction content (0.05–2.00 mm diameter %); *m*silt is the silt fraction content (0.002–0.05 mm diameter %); mc is the clay fraction content (< 0.002 mm diameter %); and orgC is the soil organic carbon (SOC) content(%).K values for each sampling point were computed by importing into GEE. These points were then interpolated to a raster layer representing spatial soil erodibility.

### Slope length and steepness factor (LS)

LS is a measure of the topographic effect in the RUSLE model. It is a function of two components, slope length (L) and slope steepness factor (S)^80^. Slope length is the horizontal distance from the origin of the erosion to the point where the slope gradient decreases, and overland flow concentrates. The LS factor was computed (Eq. 8) in GEE from the Shuttle Radar Topography Mission (SRTM V3) product for slope and the MERIT Hydrograph dataset (“MERIT/Hydro/v1_0_1″) for flow accumulation^83^.

The integration of these two datasets was based on the assumption that SRTM (30 m resolution) captures localized, fine-scale slope steepness highly accurately, whereas MERIT Hydro (~ 90 m resolution) is heavily hydrologically conditioned, making it superior for mapping continuous flow pathways and accumulation. To resolve the resolution discrepancies within GEE and minimize scaling errors in topographic indices^72^, the MERIT flow accumulation raster was resampled to match the 30 m spatial resolution of the SRTM DEM using bilinear interpolation.

8 $$\:\mathrm{L}\mathrm{S}={\left(\frac{\mathrm{F}\mathrm{l}\mathrm{o}\mathrm{w}\mathrm{A}\mathrm{c}\mathrm{c}\mathrm{*}\:\mathrm{C}\mathrm{e}\mathrm{l}\mathrm{l}\mathrm{S}\mathrm{i}\mathrm{z}}{22.13}\right)}^{0.4}\mathrm{*}{\left(\frac{\mathrm{sin}\left(\mathrm{S}\mathrm{l}\mathrm{o}\mathrm{p}\mathrm{e}\mathrm{*}\:0.01745\right)}{0.0896}\right)}^{1.3}$$

where FlowAcc is flow accumulation, Cell Size is DEM resolution, and Slope is expressed in degrees.

### Cover management factor (C)

The C factor represents the effect of vegetation cover, crop management, and surface residues on soil erosion^80^. To compute the C factor using the exponential method, Sentinel-2 imagery is first collected and preprocessed to calculate NDVI from the red and near-infrared bands (9).

9 $$\:NDVI=\frac{\mathrm{N}\mathrm{I}\mathrm{R}-\mathrm{R}\mathrm{E}\mathrm{D}}{NIR+RED}$$

The C factor is then derived using an exponential formula (10), which assigns low values for dense vegetation and high values for bare soil, effectively capturing the nonlinear effect of vegetation on soil erosion^3^. The resulting C factor map, ranging from 0 to 1, can be validated against land cover data and used in RUSLE/USLE models to estimate soil loss^71^.

10 $$\:\mathrm{C}=\mathrm{e}\mathrm{x}\mathrm{p}[-\alpha\:.\frac{NDVI}{\beta\:-NDVI}]$$

Where: NDVI ranges from − 1 to + 1, but for vegetation, usually 0 to 1. α and β are empirical constants (often α = 2, β = 1 for simple models). Higher NDVI values corresponded to lower C factors, reflecting denser vegetation cover.

### Support practice factor (P)

The P factor represents the impact of human conservation practices on reducing soil erosion by water^80^. It is defined as the ratio of soil loss with a given practice to the corresponding loss without any practice. To ensure consistency in P-factor assignment, a dual approach combining fixed land-cover values and dynamic slope-based values was applied. In this study, the P-factor map was developed by integrating the classified LULC map with the DEM-derived slope raster in GEE. First, land cover was reclassified into two primary domains: cropland and non-cropland^55^. For all non-cropland areas (e.g., forest, shrubland ), a fixed P-value of 1.0 was assigned, reflecting the absence of structural agricultural conservation measures. Conversely, for croplands, P-values were dynamically assigned according to slope percentage classes, assuming the implementation of contour tillage practices followingWischmeier & Smith^80^(Table 1). While systematic field validation was not conducted, this assumption aligns with the standard, traditional farming practices widely utilized by smallholder farmers in the Ethiopian highlands to manage runoff^30^; Hurni et al., 2015). This methodology ensures that conservation parameters are applied exclusively to agricultural areas where they naturally occur, consistent with previous studies^21^.

**Table 1:** Slope classes for assigning P value for farmlands.

Slope in percentage	*P* factor values
0–5	0.10
5–10	0.12
10–20	0.14
20–30	0.19
30–50	0.25
50–100 Other land uses	0.33 1

### Hotspot analysis

Following the RUSLE annual soil loss (A) calculation in Google Earth Engine (GEE), the resulting continuous raster data was imported into a GIS environment (such as ArcGIS) for detailed classification and visualization. Within the GIS platform, the raster was classified into a detailed hierarchy of erosion risk categories: low, moderate, high, very high, severe, very severe, and extreme to delineate areas of varying severity^57,60^. The quantitative threshold of 20 t/ha/yr served as the critical boundary for conservation strategy^31,57^. Soil loss less than 20 t/ha/yr was categorized as low-to-moderate risk. Loss greater than 20 t/ha/yr is designated a critical erosion hotspot, requiring prioritized intensive structural and biological interventions.

### Comparative consistency of model outputs

One of the persistent challenges for the RUSLE model, and many soil erosion models generally, is the difficulty in formally validating their outputs due to the scarcity of easily available, measured soil erosion records, especially in data-sparse regions^9^. Given the lack of specific measured data for the study area, full empirical validation was not feasible. Instead, the reliability of the model outputs was evaluated through a comparative consistency analysis. This involved checking the estimated soil loss rates against the results of published soil erosion studies conducted in similar watersheds or at larger regional scales within Ethiopia^51^. This method of cross-study comparison is a commonly applied approach in soil erosion literature to evaluate model estimates when direct validation data are unavailable^38,45^.

### Sensitivity analysis of RUSLE factors

The identification of the most sensitive characteristics for targeted treatments was achieved through a spatial factor exclusion (map removal) sensitivity analysis. Originally formalized for spatial overlay models^52^, this approach is highly effective for quantifying the relative contribution of individual parameters in GIS-based assessments^58^. A baseline soil loss scenario ( A_base_​) was first established using all five normal RUSLE factors. Because RUSLE is a multiplicative model^9^, each factor (R, K, LS, C, and P) was subsequently excluded one at a time(OAT) by replacing its raster layer with a neutral constant value of 1. During the exclusion of a target variable, the other four parameters were held constant to isolate the specific influence of the removed variable^28^. The resulting maximum increase and decrease in soil loss relative to the baseline were then plotted to construct the tornado chart, visually ranking each factor’s impact^10,22^.

## Result and discussion

### Agro ecology and slope of the watershed

**Fig. 5:**
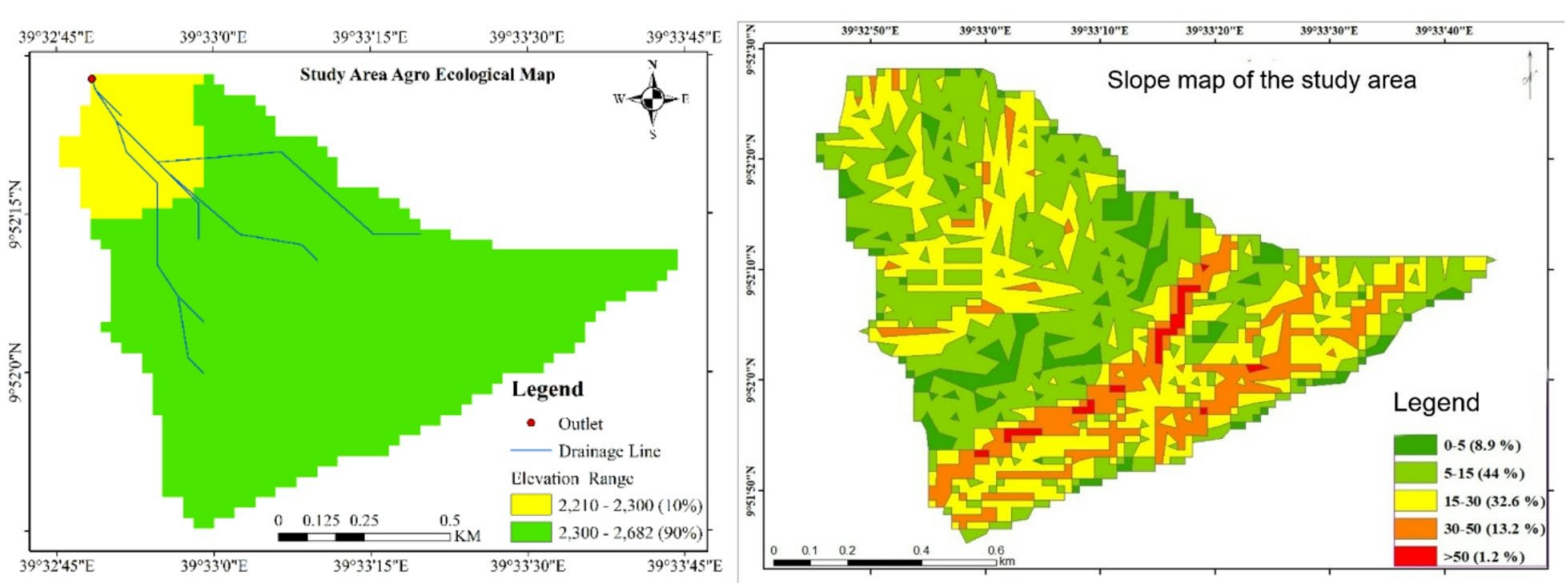
Elevation and Slope maps of the study area.

The initial biophysical characterization established the watershed’s climatic and topographic setting. Agro-ecology classification derived from DEM revealed that Megele Washa Watershed is overwhelmingly dominated by the high altitude influence Dega zone, covering 90% (104.4 ha) of the area, and the remaining 10% (11.6 ha) was mostly the outlet part of the watershed, characterized as Weyna Dega agro ecology (Fig. 5). This Dega dominance is fundamental for guiding climate-appropriate crop selection and management interventions, as Dega conditions dictate a specific growing season and specific suitability(FAO,^23^.

Slope analysis, generated from the DEM within the ArcGIS environment, confirmed the watershed’s high potential for gravitational processes and water erosion. A significant portion, 47% of the study area, falls into the steep and very steep categories (slopes above 15%) (Fig. 5). This finding is critical as slope is a primary topographic factor in the RUSLE model (LS-factor) that controls runoff velocity and erosion susceptibility. Alarmingly, the cross-tabulation of slope and land use (Table 2) showed that 49% (26.9 ha) of this inherently steep land (> 15%) is actively under cultivation. Cultivating such severe slopes greater than 15% is a primary driver of land degradation, as it accelerates soil loss and instability, often exceeding the limits set by land capability classifications for arable farming^5^. This result pinpoints an immediate biophysical constraint that necessitates mandatory land-use conversion or stringent conservation practices.

**Table 2:** Slope classification of the cultivated land of the study area.

Slope Class	Area (ha)	Land Use	Area (ha)
0–5 (Gentle)	10.3	Cultivated land	10.6
5–15 (Moderate)	51	Cultivated land	38.4
15–30 (Steep)	37.8	Cultivated land	18.8
30–50 (V. Steep)	15.3	Cultivated land	7.2
> 50 (E. Steep)	1.4	Cultivated land	0.9
Total > 15%	54.5 ha (47%)	Total Cultivated > 15%	26.9 ha (49% of steep land)

V. Steep: Very Steep, E. Steep: Extremely Steep.

### Current land use and land cover (LULC) assessment

**Fig. 6:**
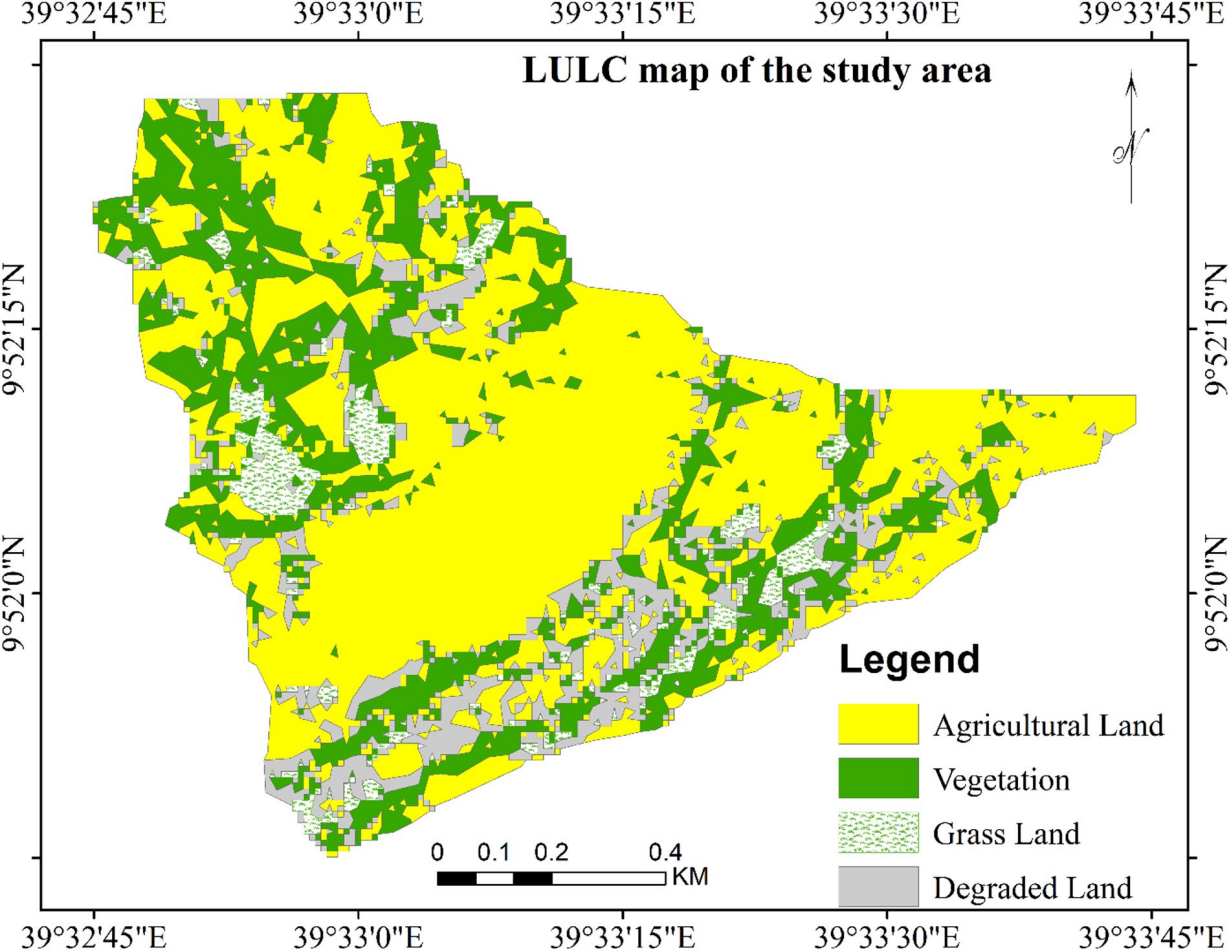
Land use and land cover maps of the Megele washa watershed.

LULC classification performed using Random Forest (RF) algorithms within GEE on Sentinel 2 imagery quantified the dominant anthropogenic pressures driving degradation (Table 3; Fig. 6). Agricultural land is the dominant land use type, covering 58.1% of the watershed. This intensive use of land for agriculture requires that future management be strictly aligned with soil-saving practices. This LULC is also a critical input for the degradation prioritization process, as the classified areas are used to derive the RUSLE C-factor (Cover-Management Factor), which modulates the impact of rainfall erosion.

In contrast, protective and non-productive land covers are distributed as follows: Vegetation (likely comprising forest and shrub land) is relatively high, 28.8 ha (24.9%), indicating a substantial reserve of vegetative cover that contributes to erosion protection and must be sustained. However, the alarming concentration of degradation is found in the Degraded Land class, which accounts for 13.3 ha (11.5%) of the watershed. This high proportion represents a severe loss of ecosystem function and necessitates urgent rehabilitation efforts to restore ecological health and provide essential ecosystem services^15^. This significant percentage of degraded land represents an immediate priority zone for intervention identified through the biophysical characterization.

### LULC classification accuracy

The reliability of the LULC map is a crucial dataset for accurately calculating the RUSLE C-factor, which was validated through a rigorous accuracy assessment of the GEE-based RF classification. Strata were defined according to the four major LULC classes: Agricultural, Vegetation, Grassland, and Degraded land. Within each class, reference points were collected in the field using GPS. The number of samples per class was proportional to its spatial extent: Agricultural (52), Vegetation (25), Grassland (7), and Degraded (20), resulting in a total of 104 reference points.

The confusion matrix (Table 4) summarizes the classification results. The Overall Accuracy (OA) of 87.5% and the corresponding Kappa coefficient of 0.80 indicate a strong agreement between classified and reference data. User’s Accuracy (UA) ranged from 71.43% (Grassland) to 96.15% (Agricultural), and Producer’s Accuracy (PA) ranged from 86.21% (Agricultural) to 100% (Grassland). These high values demonstrate that the LULC map is spatially accurate and highly reliable for subsequent quantitative modeling of soil degradation, including estimation of the RUSLE C and P factors.

**Table 3:** Land use and land cover classification and area proportions of the watershed

Land use	Area(ha)	Ratio (%)
Cultivated land	67.3	58.1
Degraded land	13.3	11.5
Vegetation	28.8	24.9
Grassland	6.4	5.5

**Table 4:** Confusion matrix and classification accuracy assessment for LULC of the Watershed

Reference / Classified	Agricultural	Vegetation		Grassland	Degraded	Row Total		Producer’s Accuracy (%)	
Agricultural	50	2		2	4	58		86.21	
Vegetation	2	21		0	1	24		87.5	
Grassland	0	0		5	0	5		100.00	
Degraded	0	2		0	15	17		88.24	
Column Total	52	25		7	20	104			
User’s Accuracy (%)	96.15	84		71.43	75				

### Soil Physico-chemical assessment across the watershed

The Soil Quality Assessment was conducted by analyzing the physicochemical properties of 90 collected samples, the descriptive statistics of which are presented in Table 5. These properties are crucial inputs for estimating the K-factor (Erodibility) in the RUSLE model and defining the fertility constraints of the watershed.

**Table 5:** Descriptive statistics of soil properties across the watershed.

Parameters	Min	Max	Mean	Median	Std. Dev.	CV (%)	Skewness	Kurtosis	Std. Error
PH	5.87	7.90	6.86	6.77	0.45	0.07	0.50	-0.28	0.05
EC	0.02	0.21	0.11	0.10	0.06	0.50	0.42	-0.16	0.01
BD	1.01	1.34	1.17	1.17	0.12	0.11	-0.15	-0.69	0.02
CEC	23	35	29.24	28.97	3.21	0.11	0.30	-0.06	0.34
ExNa	0.20	0.68	0.45	0.43	0.11	0.24	0.28	-0.31	0.01
ExK	0.12	1.65	0.86	0.84	0.32	0.37	0.21	-0.20	0.03
Ca	13.72	29.74	20.25	20.30	3.41	0.17	0.34	-0.28	0.36
Mg	0.58	11.98	5.71	5.63	2.19	0.38	0.08	0.29	0.23
OC	0.33	2.3	1.42	1.50	0.59	0.42	-0.08	-0.43	0.06
OM	0.05	4.50	2.08	1.81	0.99	0.48	0.37	-0.66	0.10
AVP	1.00	33.27	15.11	14.15	7.63	0.50	0.62	-0.29	0.80
TN	0.02	0.23	0.11	0.10	0.05	0.44	0.45	-0.36	0.01
CN_Ratio	8.70	10.30	9.56	9.58	0.28	0.03	-0.27	0.79	0.03
Sand	5.00	49.00	26.39	26.00	9.19	0.35	0.51	0.32	0.97
Clay	28	60	49.14	50.00	10.62	0.22	-0.54	0.53	1.12
Silt	6.00	38.00	23.84	24.00	5.51	0.23	-0.29	0.95	0.58

### Soil physical properties analysis

The watershed’s soils are predominantly clayey, with the textural analysis showing a mean clay content of 49.14%, silt at 23.84%, and sand at 26.39%. The high variability in sand content (CV = 35%) strongly influences infiltration rates, water holding capacity, and erosion risk, which suggests localized differences in texture^42^. The dominance of clay indicates good intrinsic nutrient retention but also implies a risk of poor drainage and structural issues, especially under compaction. Bulk Density (BD) exhibits a mean of 1.17 g/cm³ and low variability (CV = 11%), which is generally favorable for root penetration and water movement. However, the presence of higher BD values (up to 1.34 g/cm³) is likely a consequence of decreased organic matter and compaction, an outcome often associated with the intensive cultivation observed on these clay-rich soils^43^. Compaction fundamentally alters the soil architecture by reducing total porosity and compressing functional macropores into less permeable micropores. This disruption of the pore structure severely limits water infiltration and soil moisture retention capacity, which consequently accelerates surface runoff generation and exacerbates erosion risks during rainfall events^74^.

### Soil chemical properties analysis

The chemical analysis reveals key insights into current soil fertility. Soil pH is near-neutral (mean 6.86), which is optimal for nutrient availability^78^. With electrical conductivity (EC) well below the salt stress threshold, the soils are classified as non-saline^35^; yet, the high EC variability (CV = 50%) suggests localized salinity gradients that require spatial monitoring. The soil also demonstrates strong intrinsic fertility, evidenced by a high cation exchange capacity (CEC; mean 29.24 cmol(+)/kg). Conversely, available phosphorus (Av.P) shows very high variability (CV = 50%), pointing to widespread but localized P deficiencies across the area.

Despite these positive baseline indicators, the most critical indicator of soil degradation is the depletion of organic pools. Organic Carbon (OC) (mean 1.42%) and Total Nitrogen (TN) (mean 0.11%) are critically low with high spatial variability (CV ≈ 42–44%), signaling severe depletion driven by intensive farming and erosion^42^. While undisturbed SOC is protected within soil aggregates and clay complexes, continuous cultivation physically fractures these macro-aggregates, exposing organic matter to rapid microbial decomposition. Furthermore, vegetation removal leaves these structurally weakened soils vulnerable to runoff-driven water erosion, which strips away SOC-rich topsoil. This structural collapse and poor biomass return drive the rapid loss of OC and TN. Halting this degradation requires targeted revegetation to disrupt erosive flow paths and restore physical SOC stabilization mechanisms^84,85,88^.

Moreover, sodicity is not a concern in the area based on Exchangeable Sodium (ExNa) analysis. However, high variability in Exchangeable Potassium (ExK) (CV = 37%) and Magnesium (Mg) (CV = 38%) suggests that site-specific management is necessary to maintain nutrient balance because of its inconsistent availability^35^. Overall, while intrinsic properties are favorable, the depletion and high variability of OC, TN, and Av. P signifies an urgent need for Integrated Soil Fertility Management (ISFM) to combat soil quality degradation.

**Fig. 7:**
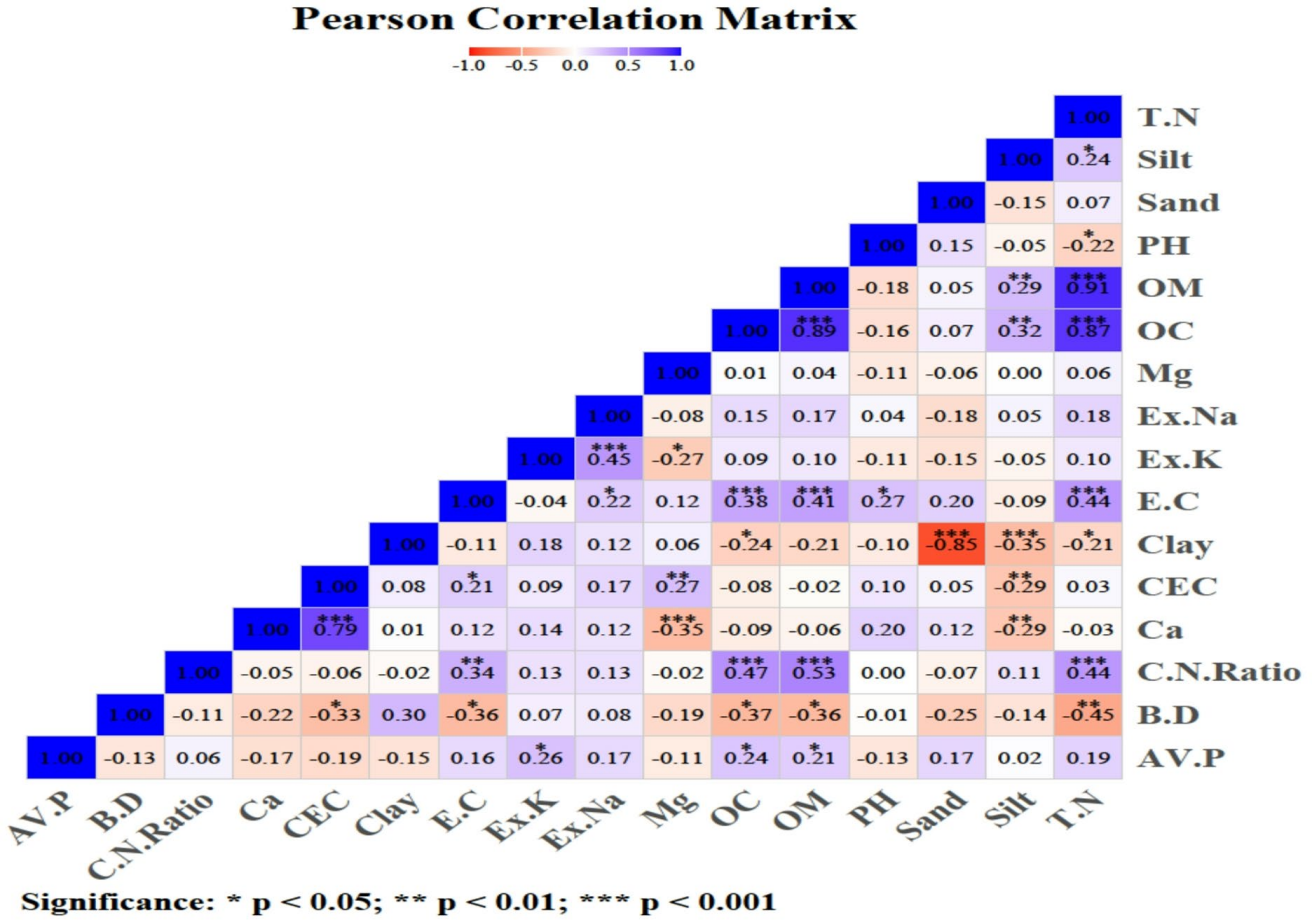
Pearson correlation matrix showing relationships among soil properties.

### Soil parameters correlation analysis

The correlation analysis (Fig. 7) measures the strength and direction of the relationship between two/ more soil parameters, which range from − 1 (perfect negative correlation) to + 1 (perfect positive correlation). The strongest positive correlations confirmed the fundamental link between key organic fertility indicators: Total Nitrogen (TN) exhibited a strong association with Organic Matter (OM) (*r* = 0.91) and Organic Carbon (OC) (*r* = 0.87), underscoring the vital role of the organic pool as the primary nitrogen reservoir^78^.

Crucially for identifying degradation mechanisms, between Bulk Density (BD) and the organic complex (TN (*r* = − 0.45), OM (*r* = − 0.36), and OC (*r* = − 0.37), the strong negative correlations were observed that strongly suggest soil compaction (high BD). It is a significant physical degradation mechanism that reduces the soil’s capacity to store organic matter and nitrogen^35^. Other notable associations included a strong positive correlation between CEC and Calcium (Ca) (*r* = 0.79), reflecting the base cation contribution to nutrient exchange, while moderate correlations between OC/OM and the C: N ratio (*r* ≈ 0.50) indicated the influence of carbon on decomposition dynamics^42^.

### Spatial modeling of selected soil properties

#### Geostatistical preprocessing and validation

**Fig. 8:**
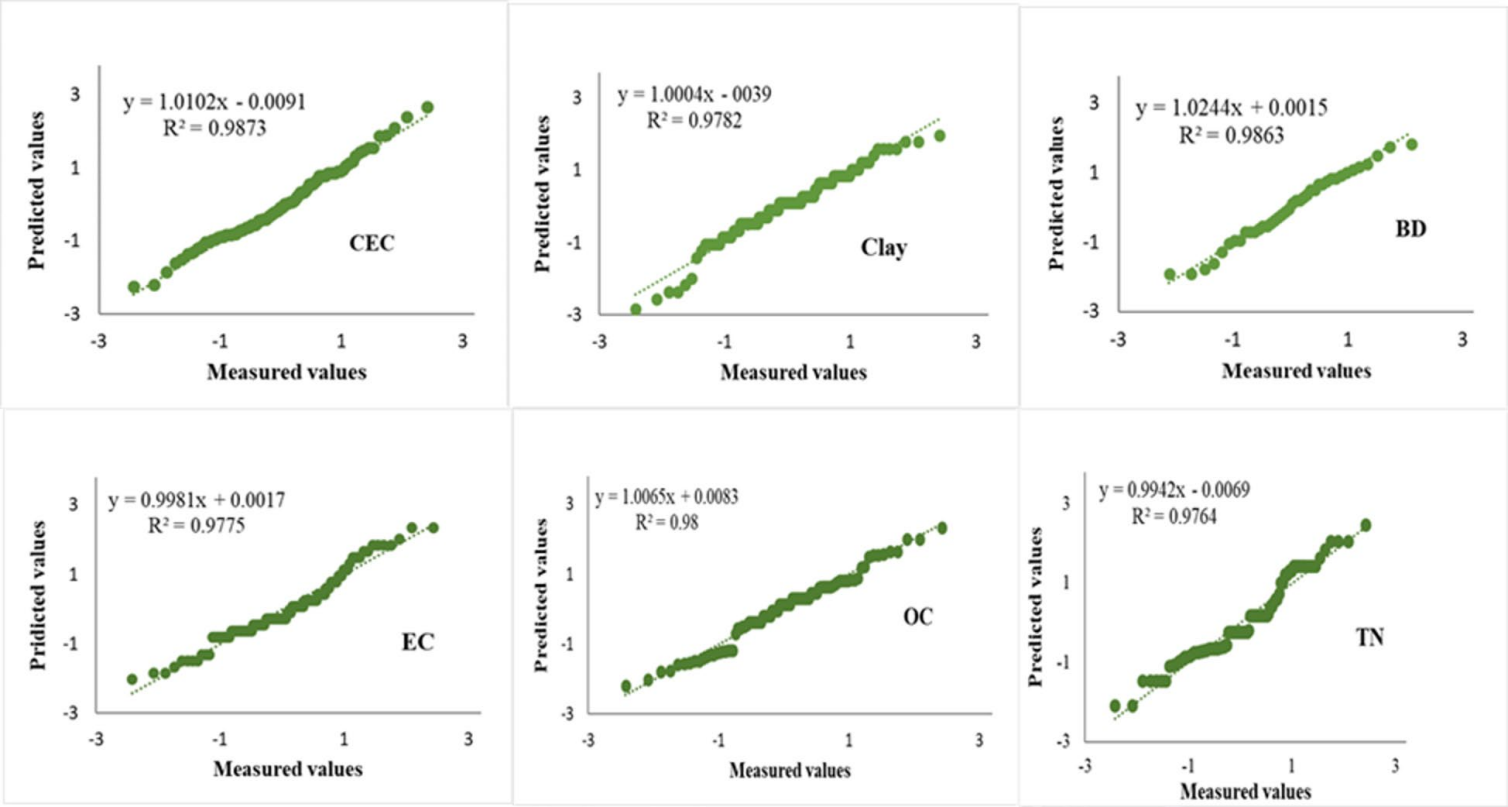
QQ plot for selected soil properties.

Organic Carbon (OC), Total Nitrogen (TN), Bulk Density (BD), Cation Exchange Capacity (CEC), Clay fraction, and Electrical Conductivity (EC) were selected for spatial modeling. Parameters were prioritized intentionally with high agronomic relevance as key indicators of soil fertility and structure (Bulk density and Clay) while minimizing redundancy (multicollinearity) based on correlation analysis^32,77^. This exclusion of highly correlated variables ensures stable model results that accurately capture the true spatial variability. The data were then validated for OK suitability. Normal Quantile–Quantile (QQ) plots (Fig. 8) confirmed that the selected parameters adequately approximated a normal distribution, with points aligning near the 45∘ line. This confirmation justified the use of Kriging for statistically sound spatial predictions.

**Fig. 9:**
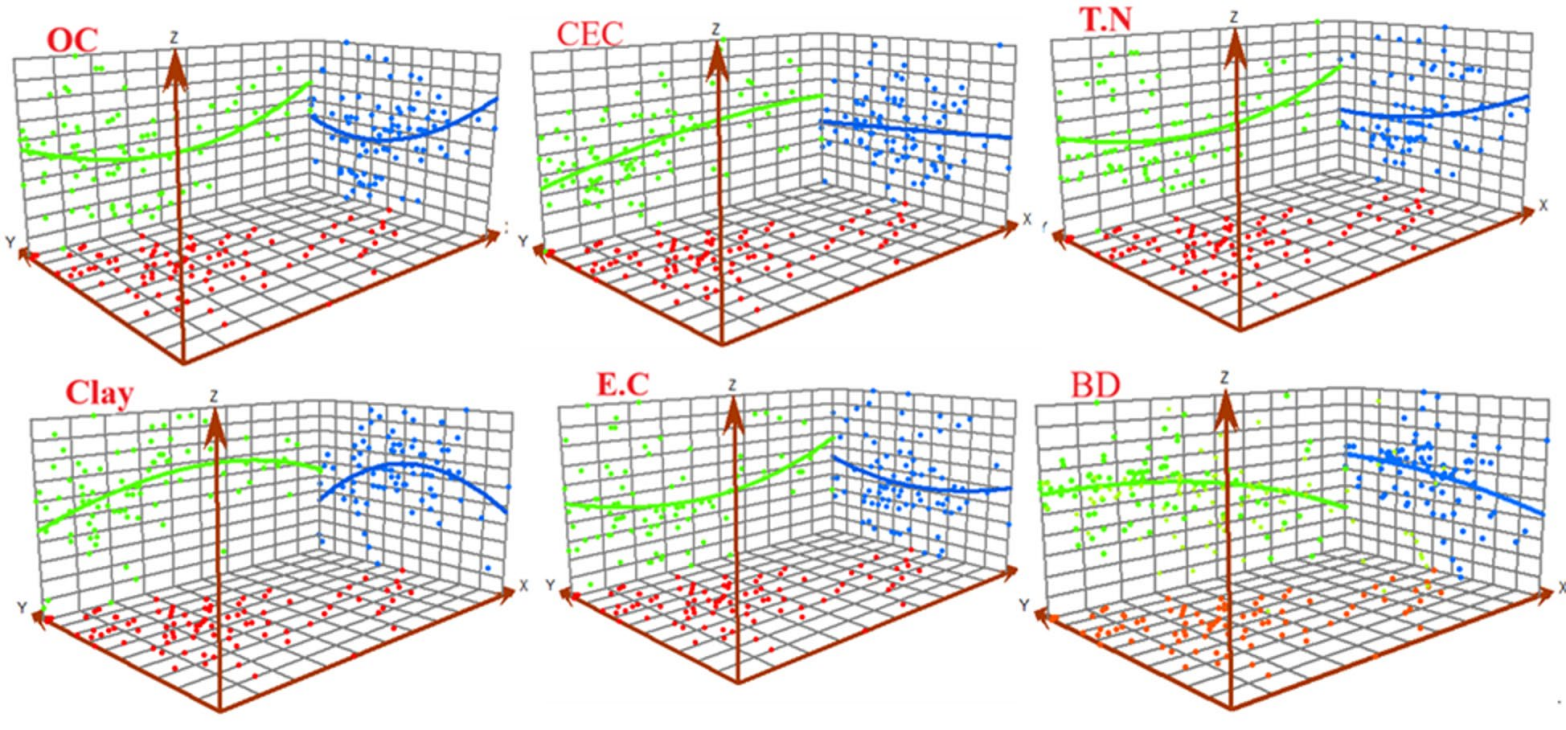
Spatial Trend Analysis Indicators of Selected Soil Properties.

Subsequently, Trend analysis (Fig. 9) revealed significantly large-scale spatial patterns that violate the stationarity assumption of OK. Specifically, OC, TN, and EC showed an increasing trend in both the East-West and North-South directions. Conversely, Clay and BD showed a decreasing trend in both directions, and CEC decreased East-West but showed no trend North-South. To isolate the local spatial variation, all soil parameters were mathematically detrended (trend removed) using first- and second-order polynomials^77^.

### Semivariogram analysis and spatial dependence

**Fig. 10:**
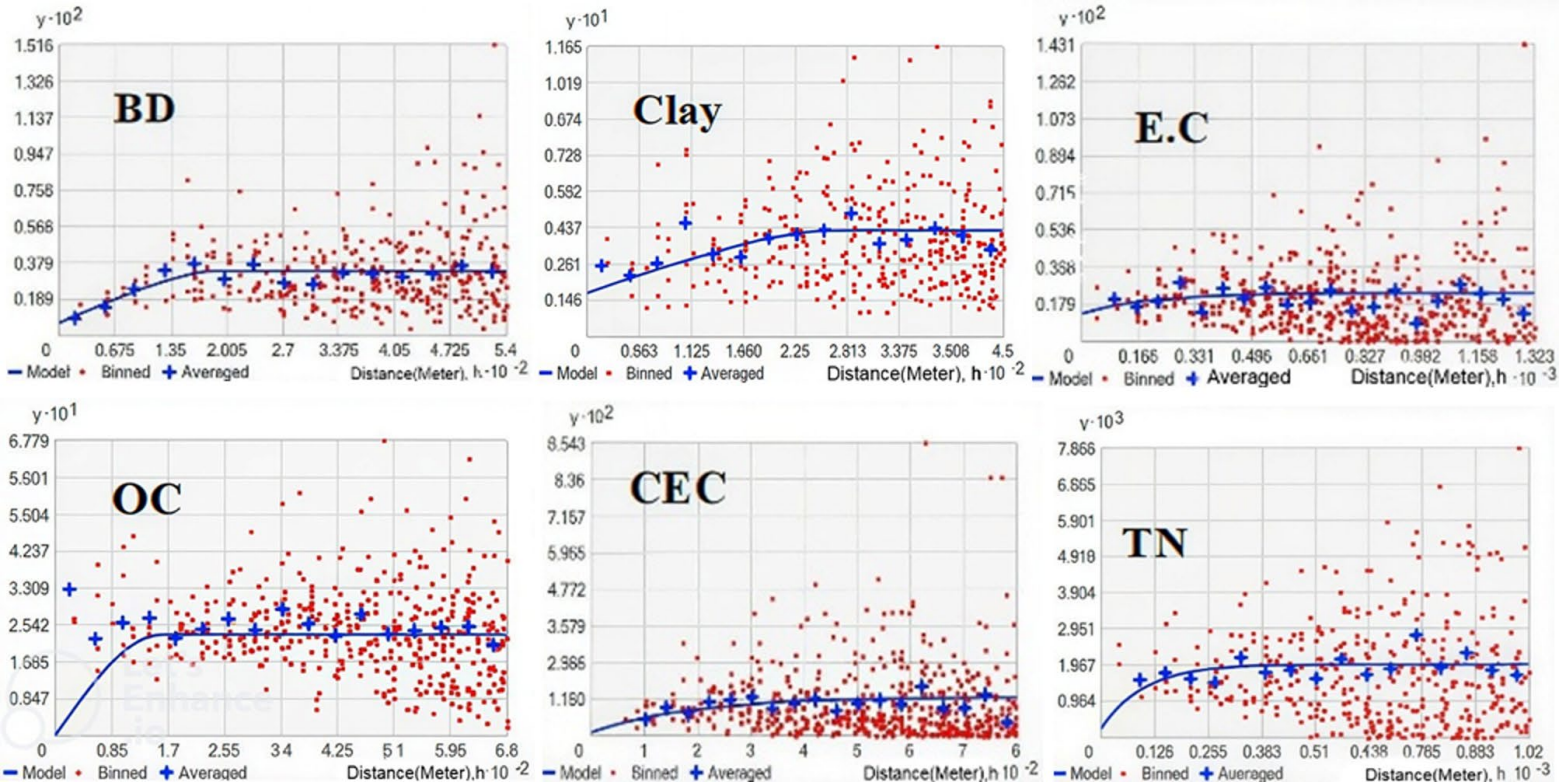
Semi-viogram maps for selected soil parameters.

Semivariogram analysis quantified the spatial structure of the detrended soil properties. Different theoretical models were fitted to the experimental semivariograms to best describe each property’s spatial pattern (Fig. 10; Table 6). Circular Model: Best described by Bulk Density (BD) and Clay content. Exponential Model: Best described by Electrical Conductivity (EC), Cation Exchange Capacity (CEC), and Total Nitrogen (TN). Spherical Model: Best described as Organic Carbon (OC). These varied model fits are typical in soil science, confirming that no single theoretical model is universally applicable; the best fit is determined by the inherent spatial continuity and complexity of the property under investigation^77^.

**Table 6:** Semivariogram parameters and spatial dependence of soil properties.

Parameter	Model Fitted	Nugget (C0​)	Sill (C0​+C)	Range (a) (m)	Nugget-to-Sill Ratio (C0​/(C0​+C))	Spatial dependency
BD (g/cm3)	Circular	0.0007	0.003	184.28	0.23	Strong
Clay (%)	Circular	0.02	0.04	253.78	0.50	Moderate
EC(dS/m)	Exponential	0.001	0.002	601.23	0.50	Moderate
OC (%)	Spherical	0.002	0.23	160.89	0.01	Strong
CEC (mg/100 g)	Exponential	0.001	0.011	550.78	0.09	Strong
TN (%)	Exponential	0.0003	0.0017	305.47	0.18	Strong

The range (a), which is the distance over which observations remain spatially correlated, varied significantly. The long ranges for EC (601.23 m) and CEC (550.78 m) suggest that their spatial distribution is governed by broader factors like geomorphology or parent material. In contrast, the shorter ranges for OC (160.89 m) and BD (184.28 m) indicated that higher local variability, likely driven by micro-topography and immediate management practices (Cambardella et al.,1994).

The strength of spatial dependence was classified using the nugget-to-sill ratio. OC (0.01), CEC (0.09), TN (0.18), and BD (0.23) all exhibited strong spatial dependence (ratio ≤ 25%). This means their variability is predominantly controlled by intrinsic soil properties such as texture and organic matter. In contrast, Clay (0.50) and EC (0.50) showed moderate spatial dependence (ratio between 25% and 75%), suggesting that their variability is also significantly influenced by extrinsic factors like cultivation and fertilizer application^76^.

### Kriging model validation

The adequacy of the selected variogram models was thoroughly assessed using leave-one-out cross-validation (Table 7). Cross-validation statistics supported the adequacy of the selected models. The root mean square standardized error (RMSSE) values were close to 1 (0.97–1.17), confirming unbiased predictions. Except OC, the relatively low root mean square error (RMSE) as compared from10-20% of data range values^14^ further indicates reliable model performance. Overall, the results demonstrate that most soil properties exhibited structured spatial dependence with appropriate variogram models and meaningful ranges, confirming the suitability of geostatistical techniques for spatial prediction in the study area.

**Table 7:** Ordinary Kriging cross-validation metrics for selected soil properties.

Parameter	Root Mean Square Error (RMSE)	Root Mean Square Standardized Error (RMSSE)
BD (g/cm3)	0.058	1.17
Clay (%)	4.56	1.04
EC(dS/m)	0.036	1.02
OC (%)	0.59	0.97
CEC (mg/100 g)	2.2	1.09
TN (%)	0.02	1.02

**Fig. 11:**
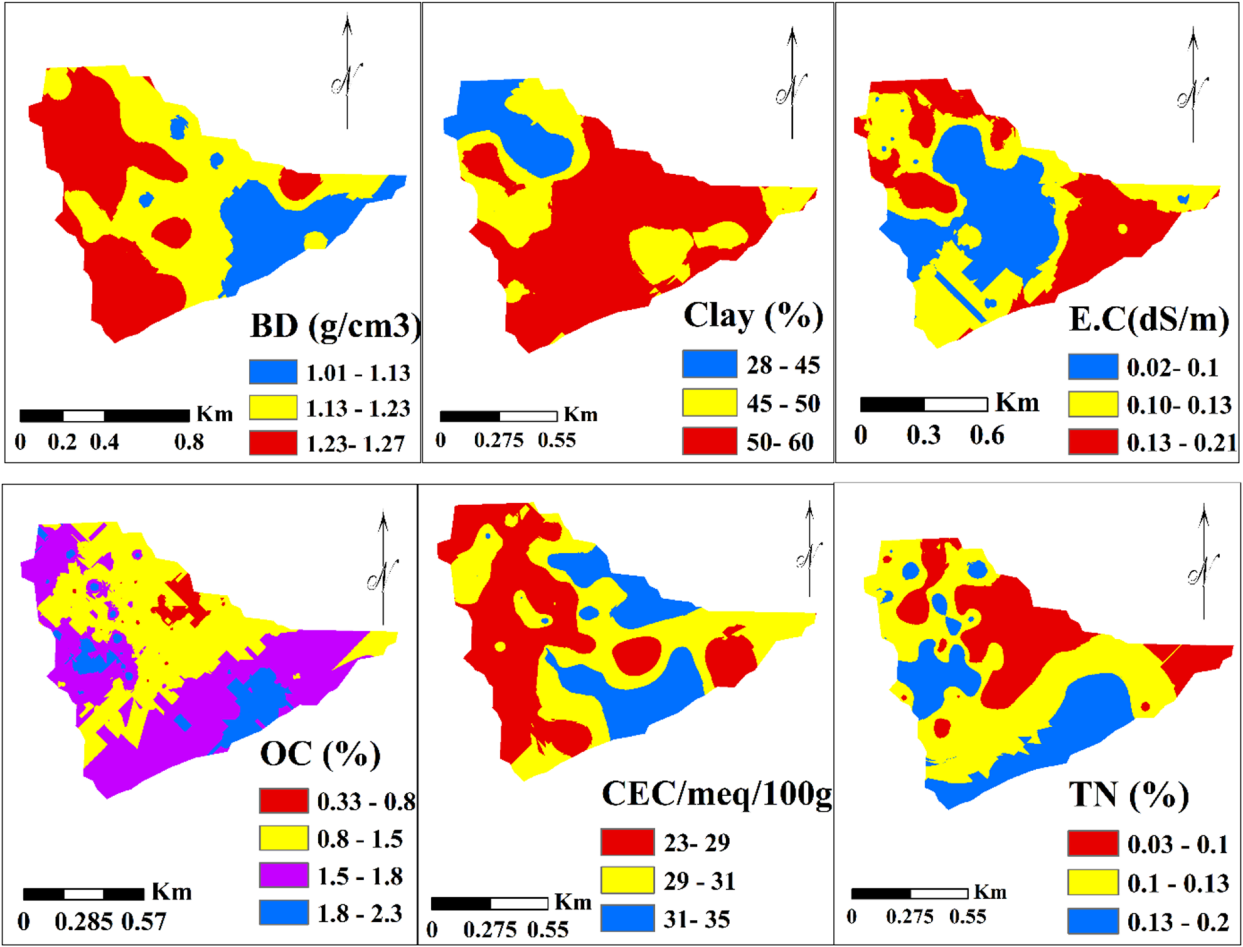
Spatial prediction maps of selected soil properties.

The spatial prediction maps (Fig. 11) show distinct patterns of soil health and degradation across the study watershed. Physical degradation is indicated by Bulk Density, which shows its highest values (compaction hotspots) in the western and North-West parts, mainly due to the intensive irrigation associated with horticulture in the study area^29^. In contrast, the lowest BD was observed mainly in the southern zone of mostly vegetative area and the Middle section of frequent conventional tillage in agricultural lands^78^.

Organic Carbon (OC) and Total Nitrogen (TN) observed in the lowest concentrations across the Middle, eastern, and Northern parts indicate the most widespread issues of fertility depletion of cultivated land. This strong spatial correlation between OC and TN confirms the widespread loss of the nitrogen pool and organic matter due to intensive cultivation and biomass removal^39,41^. In contrast, in these zones, somehow better Cation Exchange Capacity (CEC) reflects a soil with good capacity but poor current fertility, in which management practices like adding organic matter or fertilizers are needed to make that capacity productive. This depletion is also evidenced by lower CEC values in the western and Northern parts, indicating reduced nutrient holding capacity^29^.

Moreover, the watershed is under minimal salinity risk as Electrical Conductivity (EC) remains generally low, but localized higher EC in the North-West and North-East may indicate potential salt accumulation issues, which could be related to irrigation and drainage practices^1^. Across most of the watershed, the Clay Content is dominant, but is lower at the vegetation-dominated outlet. While direct sediment transport data were not collected in this study, the observed spatial variation may be associated with the redistribution of finer sediments within the watershed^78^. In summary, the watershed is geographically segmented by degradation type: compaction dominates the irrigated East/Northeast, and severe nutrient depletion affects the intensively tilled Middle and eastern zones.

### Mapping and spatial analysis of RUSLE parameters

**Fig. 12:**
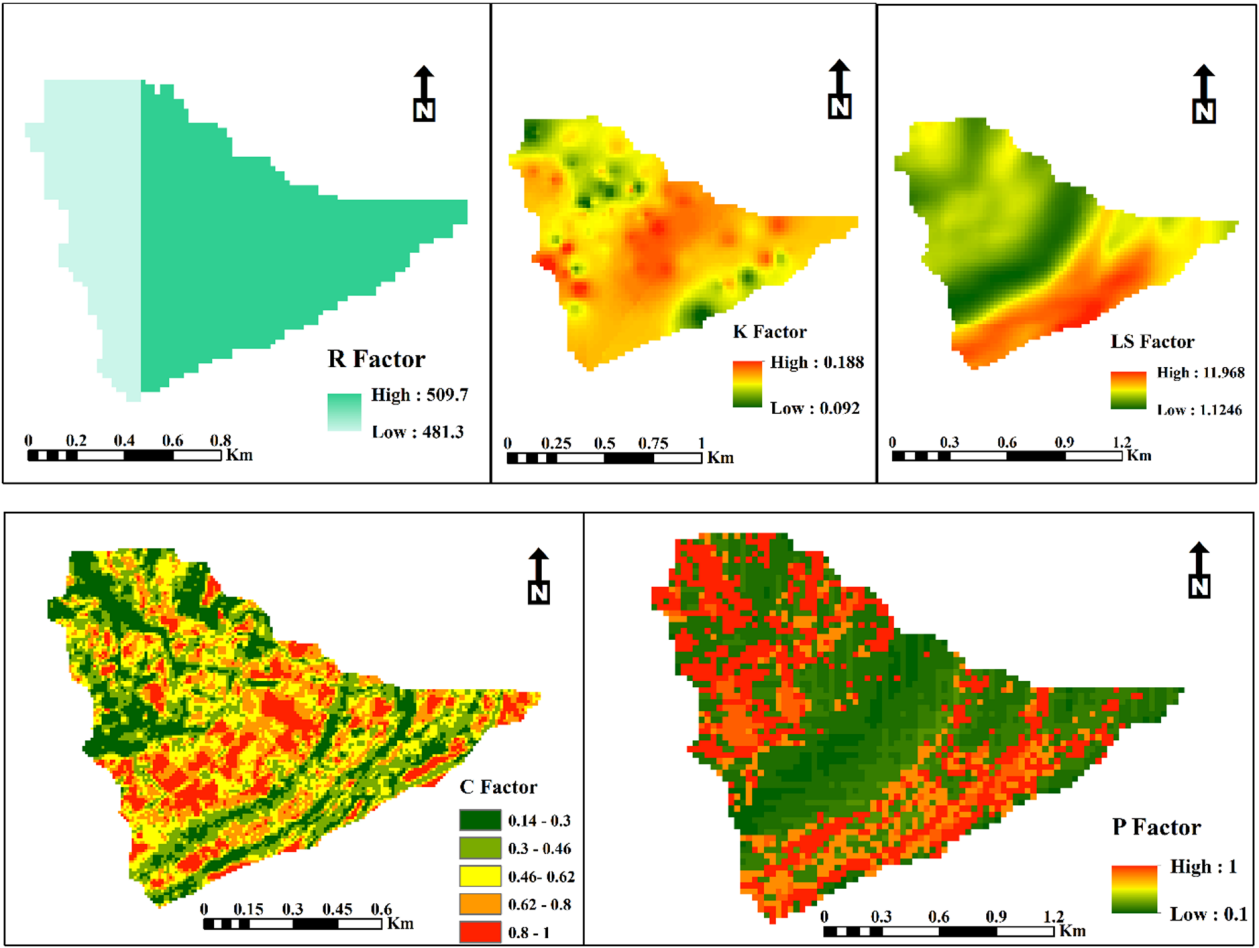
Spatial distribution maps of RUSLE parameters.

Mapping (Fig. 12) and analyzing RUSLE parameters provide a clear understanding of how climatic, topographic, and land management conditions interact to influence soil erosion across the landscape. The following sections present and discuss the spatial patterns of each RUSLE factor in detail.

### R-Factor (rainfall erosivity)

The erosive power of rainfall and runoff is quantified with the R-factor that shows a minimal range of 481.3 to 509.7 MJ mm h−1 ha−1yr − 1. This limited spatial variability suggests that rainfall is a consistently high baseline contributor to erosion across the entire watershed, with only a slight increase in erosivity observed towards the eastern part. Due to its uniformity, the R-factor is not the primary driver of the spatial differences in final soil loss, but it establishes a high inherent climatic potential for erosion throughout the study area^54^.

### K-Factor (soil erodibility)

This factor exhibits spatial variability that ranges from 0.092 to 0.188 t h MJ−1 mm − 1, with the highest erodibility values concentrated in the central-eastern portion and in scattered patches along the western boundary. While the dominant soil type in this central-eastern agricultural area is often classified as clay, which typically resists detachment, the observed high K values are a critical finding. They indicate that the K-factor is likely controlled by structural degradation from continuous cultivation, low organic matter content, and/or localized variations in silt fraction. This interpretation is further supported by the soil property maps (Fig. 11), which reveal a clear spatial correspondence between high K-values and areas characterized by low soil organic carbon (OC) and high bulk density (BD). These conditions indicate degraded soil structure, reduced aggregate stability, and increased susceptibility to erosion by rainfall^59,80^.

### Topographic factor

The LS-factor reflecting the effect of topography is a spatially variable physical factor, ranging from 1.1246 to an extreme maximum of 11.968. The highest LS values are highly concentrated along the steep southern and northern perimeter slopes. This steep topography dictates that the LS-factor is the primary physical constraint, driving significant runoff acceleration and severe erosive power in these zones^49,59^.

### C-Factor (cover management)

The C-factor shows significant spatial contrast, with values ranging from 0.14 to 1, with the highest values prevalent in the north-eastern, central, and southern perimeter areas, corresponding to the cultivated or degraded grazing lands. The lower C values are distributed in the watershed’s northern, northwestern, and southern parts over conserved forests and managed watersheds. The high-C zones represent areas where poor land management practices leave the soil most exposed to erosive forces, confirming the C-factor as a key manageable driver of erosion^54,59,80^.

### P-Factor (support practice)

The P-factor, reflecting the effectiveness of SWC practices, ranges from 0.1 to 1, with a high P-value, particularly within the areas of greatest erosion risk. Except for the middle parts of agricultural land, particularly the top parts of the watershed, which indicate a violation of the watershed management rule (starting from the top part). Moreover, the northern, northwest, and northeast parts of the bottom watershed lack appropriate conservation measures. The overall high P-factor underscores the insufficiency or absence of current conservation efforts in mitigating erosion across the vulnerable parts of the watershed^6,59,80^.

### Estimated soil loss and compared with other studies

**Fig. 13:**
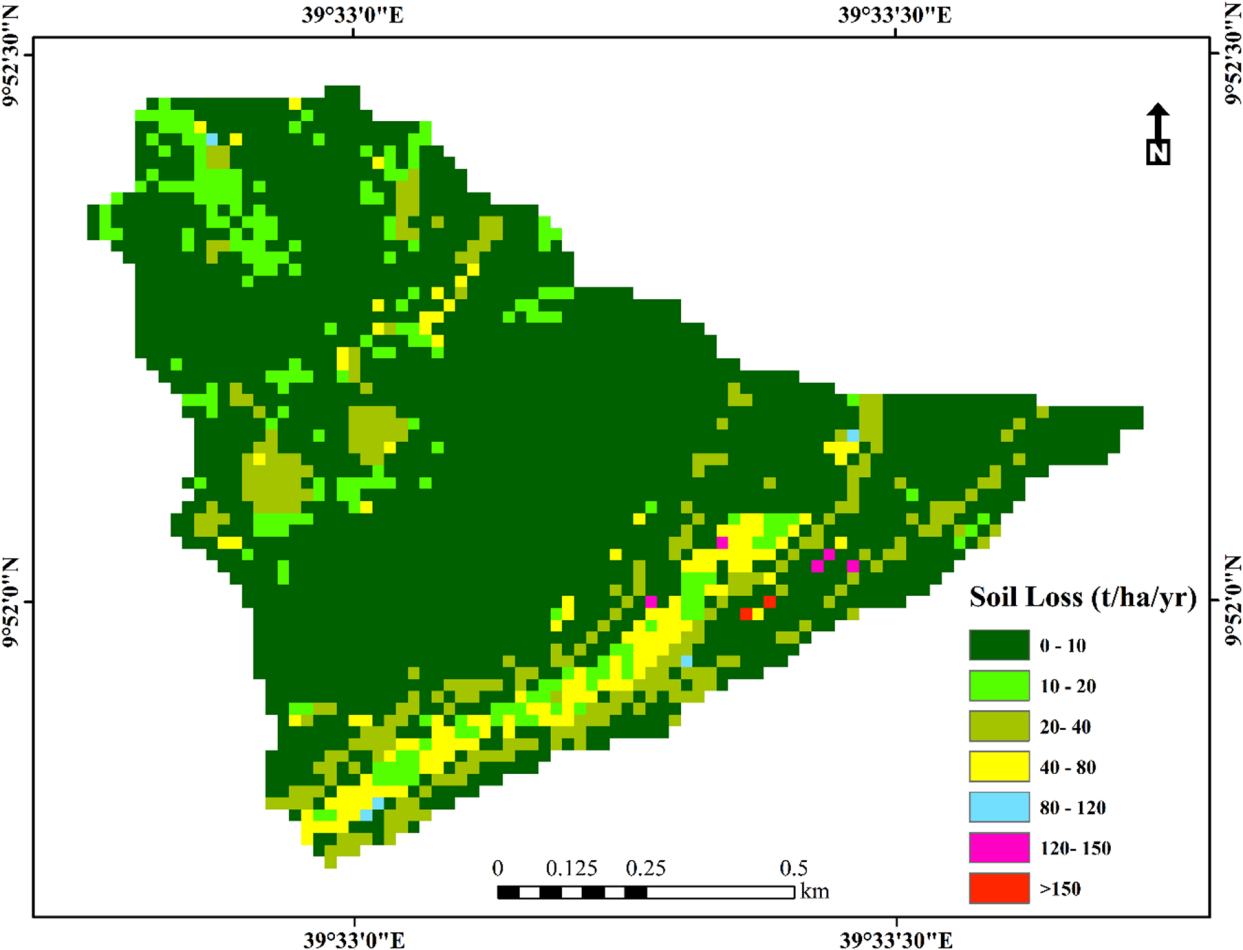
Spatial distribution of soil loss rate severity in the Megele washa watershed (0–10: Low), (10–20: Medium), 20–40: High), (40–80: very high), (80–120: severe), (120–150: very severe), (> 150: Extremely severe)

The estimated average soil loss of the study area is 26.5 t ha⁻¹ yr⁻¹, which indicates a severe erosion rate when compared to both natural soil regeneration capacities and established tolerance limits. Using the field-collected average topsoil bulk density of 1.17 g cm⁻³, this mass equates to an estimated continuous soil depth loss of approximately 2.26 mm yr⁻¹. In the Ethiopian highlands, natural soil formation rates are generally estimated to range from 2 to 11 t ha⁻¹ yr⁻¹, corresponding to a depth increase of only 0.3 to 1.1 mm yr⁻¹^33,34^. While true environmental sustainability requires soil loss to remain below these formation rates, historical agricultural management guidelines have set the Tolerable Soil Loss Limit (TSLL) slightly higher, ranging from 2 t ha⁻¹ yr⁻¹ for shallow soils up to a maximum of 18 t ha⁻¹ yr⁻¹ for deep soils^33,34^; Morgan, 2005). Therefore, with an average loss of 26.5 t ha⁻¹ yr⁻¹, the study area not only far exceeds its natural soil regeneration capacity but also surpasses the absolute maximum agricultural TSLL by approximately 47%. Additionally, the maximum estimated soil loss of 164.4 t ha⁻¹ yr⁻¹ signifies the presence of extreme erosion hotspots within the study area. As illustrated in Fig. 13, these extreme losses are typically concentrated on steep slopes subjected to high degradation and intense cultivation with minimal soil conservation measures.

Estimated soil erosion was compared with findings of severe land degradation documented across the Ethiopian highlands in numerous RUSLE-based studies to evaluate the model, which frequently report mean annual soil losses ranging from 20 t ha−1yr − 1 to over 80 t ha−1yr − 1 in similar highland watersheds, such as 22.3 t ha−1yr − 1 in Andit Tid^18^, 24.61 t ha−1yr − 1 in Katar watershed^67^, and 30.6 t ha−1yr − 1 in Domba^47^. These comparative results underscore the urgent need for targeted, intensive soil and water conservation (SWC) measures to bring the erosion rate down below the sustainable threshold of 18 t ha−1yr − 1.

### Identification and prioritization of erosion hotspots

The spatial classification of soil loss of the watershed shows that 78% of the area falls into the low-to-moderate risk categories (0 − 20 t ha−1yr − 1). This indicates that the majority of the land is relatively stable or requires basic conservation measures^59^. In contrast, highly degraded, falling into the High, Very High, Severe, Very Severe, and Extreme Severe classes (20 t ha−1yr − 1 and above), shared 22% of the watershed area. This highly concentrated area, though smaller in extent, contributes disproportionately to the total annual sediment yield and represents the critical erosion hotspots where the maximum soil loss of 164.4 t ha−1yr − 1 occurs.

This spatial breakdown is vital for conservation prioritization, suggesting that resources should be strategically targeted^57^. 78% area needs sustainable agricultural and maintenance practices to prevent future degradation, while the 22% area demands immediate, intensive structural and biological interventions (such as terracing and exclosures) to effectively mitigate the severe land degradation and bring the overall soil loss rate down toward the tolerable limit^31,57,59^.

### Sensitivity Analysis of RUSLE Factors

**Fig. 14:**
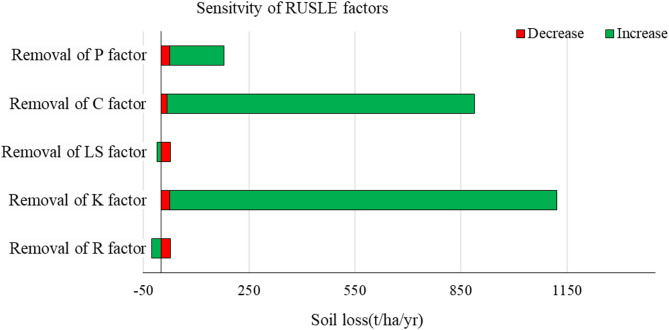
Sensitivity index of RUSLE factors in the Megele Washa watershed.

The soil erodibility factor (K) was identified as the most sensitive variable within the studied area, as depicted in Fig. 14. This observation indicates that soil attributes, including texture, structure, and organic matter content, play a pivotal role in determining erosion potential^59^; Prasannakumar et al., 2012). Following the K factor, the C-factor and P-factor illustrate the critical importance of vegetation cover and conservation practices in mitigating soil loss (Karthick & Periyasamy, 2017; Zeleke and Hurni, 2001). This finding aligns with prior research, which highlights that soil characteristics and land management strategies exert a more substantial influence on erosion dynamics compared to climatic or topographical factors (Diwediga et al.^19^; Zeleke and Hurni, 2001; Minervino et al., 2023). The analysis emphasizes the necessity for prioritizing soil quality enhancement and augmentation of vegetation cover as integral components for effective erosion control and sustainable land management within the Megele washa watershed.

### Limitations and uncertainties

While this study provides valuable insights into the spatial distribution of soil erosion, several limitations must be acknowledged. Foremost, the scarcity of field-measured soil loss data in the study area precluded empirical validation; thus, the model outputs represent estimates of comparative consistency rather than absolute measured values^7^. Additionally, as an empirical model, RUSLE is restricted to estimating sheet and rill erosion and does not account for gully or stream-bank erosion, which may lead to an underestimation of total watershed soil loss^2,59^. Furthermore, the accuracy of these estimates is inherently constrained by the resolution of the input datasets. The use of a 30-meter Digital Elevation Model (DEM) can smooth micro-topographic features and steep slope gradients, potentially yielding conservative estimates for the topographic (LS) factor^48,82^. Finally, the 30-meter Land Use/Land Cover (LULC) imagery may contain mixed pixels that obscure small-scale vegetation changes or localized agricultural conservation practices, affecting the precision of the C and P factors^73^. Future research should prioritize localized field monitoring to continuously calibrate and refine these spatially distributed estimates.

## Conclusion and recommendation

In conclusion, the Biophysical Characterization reveals the structural constraints, showing that 47% of the watershed is inherently constrained by steep slopes (> 15%) within the Dega agro-ecology. The Land Use/Land Cover (LULC) analysis confirms the functional failure, showing that an alarming 49% of this steep, vulnerable land is actively under cultivation, with agricultural dominance also contributing to the 11.5% classified as severely degraded land. This land-use practice is the primary, human-driven accelerant of the erosion crisis. The Soil Quality Assessment concurrently confirms widespread fertility decline, evidenced by critically low levels of Organic Carbon (OC) and Total Nitrogen (TN). This indicates that the soil’s productive capacity is actively collapsing in cultivated zones. This is quantitatively proven by the Soil Erosion Estimation (RUSLE), which shows the average annual soil loss for the entire watershed is 26.5 t ha−1yr − 1, a rate that significantly exceeds the tolerable limits and peaks locally at 164.5 t ha−1yr − 1. In addition, the spatial analysis tells us that this severe loss is concentrated in the critical erosion hotspots, which comprise 22% of the watershed (≥ 20 t ha−1yr − 1). These findings suggest that immediate and intensive conservation efforts must be spatially concentrated on this 22% hotspot area. Moreover, soil erodibility factor (K) was found to be the most sensitive, followed by c and p, which indicates the need for soil quality improvement, enhancing vegetation cover, and conservation practices, especially in the top parts of the watershed.

Therefore, to effectively reduce erosion and reverse fertility decline, this study recommends a dual approach. First, the conversion of highly susceptible steep agricultural lands to protective vegetation cover is essential, prioritizing the establishment of fast-growing native tree species and perennial grasses to stabilize slopes and reduce runoff. Second, the large-scale implementation of Integrated Soil Fertility Management (ISFM) practices across the watershed is required to restore depleted soil nutrients, with priority given to organic amendments such as compost application and green manuring to improve soil organic carbon (OC) and total nitrogen (TN). Additionally, enhancing vegetation cover and adopting conservation measures, particularly within the identified 22% critical zones, can further reduce runoff and strengthen soil resilience.

## Data Availability

The datasets used and/or analysed during the current study are available from the corresponding author on reasonable request.
